# Association mapping of seed quality traits using the Canadian flax (*Linum usitatissimum* L.) core collection

**DOI:** 10.1007/s00122-014-2264-4

**Published:** 2014-01-26

**Authors:** Braulio J. Soto-Cerda, Scott Duguid, Helen Booker, Gordon Rowland, Axel Diederichsen, Sylvie Cloutier

**Affiliations:** 1 Department of Plant Science, University of Manitoba, 66 Dafoe Road, Winnipeg, MB R3T 2N2 Canada; 2 Cereal Research Centre, Agriculture and Agri-Food Canada, 195 Dafoe Rd, Winnipeg, MB R3T 2M9 Canada; 3 Morden Research Station, Agriculture and Agri-Food Canada, Route 100, Morden, MB R6M 1Y5 Canada; 4 Crop Development Centre, College of Agriculture and Bioresources, University of Saskatchewan, 51 Campus Drive, Saskatoon, SK S7N 5A8 Canada; 5 Plant Gene Resources of Canada, Agriculture and Agri-Food Canada, 107 Science Place, Saskatoon, SK S7N 0X2 Canada; 6 Genomics and Bioinformatics Unit, Agriaquaculture Nutritional Genomic Center (CGNA), Km 10 Camino Cajón-Vilcún, Temuco, La Araucania Chile

## Abstract

*****Key message***:**

**The identification of stable QTL for seed quality traits by association mapping of a diverse panel of linseed accessions establishes the foundation for assisted breeding and future fine mapping in linseed.**

**Abstract:**

Linseed oil is valued for its food and non-food applications. Modifying its oil content and fatty acid (FA) profiles to meet market needs in a timely manner requires clear understanding of their quantitative trait loci (QTL) architectures, which have received little attention to date. Association mapping is an efficient approach to identify QTL in germplasm collections. In this study, we explored the quantitative nature of seed quality traits including oil content (OIL), palmitic acid, stearic acid, oleic acid, linoleic acid (LIO) linolenic acid (LIN) and iodine value in a flax core collection of 390 accessions assayed with 460 microsatellite markers. The core collection was grown in a modified augmented design at two locations over 3 years and phenotypic data for all seven traits were obtained from all six environments. Significant phenotypic diversity and moderate to high heritability for each trait (0.73–0.99) were observed. Most of the candidate QTL were stable as revealed by multivariate analyses. Nine candidate QTL were identified, varying from one for OIL to three for LIO and LIN. Candidate QTL for LIO and LIN co-localized with QTL previously identified in bi-parental populations and some mapped nearby genes known to be involved in the FA biosynthesis pathway. Fifty-eight percent of the QTL alleles were absent (private) in the Canadian cultivars suggesting that the core collection possesses QTL alleles potentially useful to improve seed quality traits. The candidate QTL identified herein will establish the foundation for future marker-assisted breeding in linseed.

**Electronic supplementary material:**

The online version of this article (doi:10.1007/s00122-014-2264-4) contains supplementary material, which is available to authorized users.

## Introduction

Oil crops have gained in importance worldwide over the past 20 years as indicated by the increase in total harvested area from 189.3 million hectares in 1992 to 272.7 million hectares in 2011 (FAOSTAT 2013). This increase hinges partly on the versatility of their fatty acid profiles which play a significant role in the nutritional properties and the end-use functionality of oil crops. In this regard, linseed (*Linum usitatissimum* L.), with its high content of alpha linolenic acid, is unique. With ~23 % of world production, Canada is the world’s largest linseed producer and exporter followed by China and the Russian Federation (FAOSTAT 2013).

Linseed is an annual, self-pollinated species with a genome size of ~370 Mb (Ragupathy et al. 2011). Domesticated in the Near East 9,000 years ago (Harris 1997), linseed is considered the oldest oilseed in the world. Its seed oil (~35–50 %) is composed of five main fatty acids (FAs): palmitic (PAL; C16:0, ~6 %), stearic (STE; C18:0, ~2.5 %), oleic (OLE; C18:1, ~19 %), linoleic (LIO; C18:2, ~13 %) and linolenic (LIN; C18:3, ~55 %) (Westcott and Muir 2003; Diederichsen et al. 2013). The high percentage of LIN distinguishes it from other oilseeds in the industrial, human food and animal feed markets. Its oxidative instability, ensuing in a soft and flexible film, and the absence of volatile organic compounds (formaldehyde, aldehydes and benzene), resulting in reduced environmental hazards (Green et al. 2008), makes linseed oil valuable in industry for paints, linoleum flooring, inks and varnishes (Cullis 2007). In addition, LIN is the precursor of the long chain polyunsaturated fatty acids eicosapentaenoic acid (EPA), docosapentaenoic acid (DPA) and docosahexaenoic acid (DHA) which are synthesized in the human body and recognized for their health benefits (Simopoulos 2000).

Linseed breeders have focused mainly on maintaining the high LIN content, while PAL, STE, OLE and LIO which correlate negatively with LIN tend to be selected against (Cullis 2007). High LIN (>65 %) germplasm is available (Friedt et al. 1995; Kenaschuk 2005), but agronomic improvement of many of these sources is needed to achieve adaptability. The first high LIN linseed cultivar NuLin™ 50 was registered in Canada by Viterra (http://www.viterra.ca). Altered FA profiles in linseed, for example low LIN (2–4 %) and high LIO (>50 %) obtained by mutation breeding, has proven effective in improving the oxidative stability and suitability of linseed oil for a variety of food uses (Green et al. 2008). Green and Marshall (1984) developed linseed lines with reduced LIN content (<29 %) using ethyl methane sulfonate (EMS)-mediated mutagenesis. Further reduction in LIN content to ~2 % was later achieved (Green 1986; Rowland 1991). Fatty acid desaturase 3 genes *lufad3a* and *lufad3b* had point mutations causing premature stop codons in one of the characterized EMS mutant lines resulting in non-functional FAD3 enzymatic activity (Vrinten et al. 2005). Additional variations in FA composition, including lines with elevated OLE and PAL content, have also been developed (Green, unpublished data; Rowland and Bhatty 1990).

Various aspects of the genetic control of storage oil biosynthesis in linseed have been studied (Green 1986; Fofana et al. 2004; Sørensen et al. 2005; Vrinten et al. 2005; Khadake et al. 2009; Banik et al. 2011) and new genes such as *LuFAD2*-*2* (Khadake et al. 2009) and *fad3c* (Banik et al. 2011) encoding FA desaturases have been cloned, broadening the options for modifying linseed FA profiles for new end uses. Generally, oilseed breeding is a more complex undertaking than the breeding of cereals or legumes, as many oilseeds such as soybean, rapeseed, sunflower and linseed have the potential to be dual- or multi-purpose crops, which require the simultaneous manipulation of quality and agronomic traits (Vollmann and Rajcan 2009). Conventional breeding has been conducted in linseed for over a century and has been particularly successful in adapting crop phenology to regional growing seasons as well as providing yield stability across environments (Green et al. 2008). However, the phenotypic selection of quantitative traits, such as oil content and FA composition, is complicated by environmental effects (Cloutier et al. 2011) that significantly reduce breeding gain. In Canada, oil content can vary up to 15 % (range 35–50 %) in individual farm samples (Duguid 2009) and the percentage of LIN can be as much as 5 % higher in cool environments (Fofana et al. 2006).

Consumer awareness of oil quality is becoming an increasingly important variable that conditions shifts in the food ingredient selection process, thereby creating new market opportunities (Wilson 2012). Acceleration of breeding cycles could translate into the edge necessary to respond to these new market demands in a timely fashion.

The use of marker-assisted selection (MAS) for oil content and FA composition can improve the efficiency of traditional linseed breeding. However, MAS requires the development of genomic tools such as molecular markers and linkage maps (Cloutier et al. 2009, 2011, 2012a, b). These tools have been recently developed in linseed, establishing the foundation for the application of MAS (Roose-Amsaleg et al. 2006; Cloutier et al. 2009, 2011, 2012a, b; Deng et al. 2010, 2011; Ragupathy et al. 2011; Soto-Cerda et al. 2011a, b; Kumar et al. 2012; Wang et al. 2012).

Quantitative trait loci (QTL) mapping based on bi-parental crosses has been the most applied approach to map QTL associated with oil content and FA in crops such as rapeseed (Zhao et al. 2005; Hu et al. 2006; Qiu et al. 2006; Smooker et al. 2011), maize (Goldman et al. 1994; Wassom et al. 2008; Yang et al. 2010) and soybean (Chung et al. 2003; Bachlava et al. 2009; Qi et al. 2011; Xie et al. 2012). In linseed, however, only one QTL study related to oil content and FA composition has been carried out, positioning QTL for iodine value (IOD), PAL, LIO and LIN (Cloutier et al. 2011). While QTL mapping has been very successful in detecting QTL, the bi-parental nature of the populations often resulted in large confidence intervals for the QTL positions which, combined with a limited number of alleles at each locus, hindered their applications in MAS (Gupta et al. 2005; Ersoz et al. 2009; Myles et al. 2009).

Association mapping (AM) or linkage disequilibrium (LD) mapping has emerged as a complementary approach to QTL mapping (Myles et al. 2009). Its power relies on the utilization of a large population of individuals with a higher level of allelic diversity that improves the probability of QTL detection and the mapping resolution (Ersoz et al. 2009). AM has been useful in dissecting the complex genetic architecture of oil content and FA composition in oil crops such as rapeseed (Honsdorf et al. 2010; Zou et al. 2010), peanut (Wang et al. 2011), soybean (Li et al. 2011) and maize (Cook et al. 2012; Li et al. 2013). These AM studies not only validated previous results from QTL mapping showing the FA biosynthesis pathway similarity among oil crops, but also identified new QTL and candidate genes useful for improving oil content and quality.

In our previous study, we characterized the flax core collection of 407 accessions assembled from the Canadian flax world collection preserved by Plant Gene Resources of Canada (Diederichsen et al. 2013), and showed its abundant genetic diversity, weak population structure and familial relatedness, and a relatively fast LD decay, all positive attributes for AM studies (Soto-Cerda et al. 2013). In the present study, we conducted AM for oil content and FA composition traits on 390 accessions aiming to identify QTL underlying these seed quality traits, which could be used for accelerating linseed breeding through MAS and for identifying germplasm with desirable characteristics.

## Materials and methods

### Plant material, genotyping and field experiments

A core collection of 407 *L. usitatissimum* accessions assembled from the Canadian World collection of flax (~3,500 accessions) (Diederichsen et al. 2013) was genotyped with 460 microsatellite (SSR) markers (Roose-Amsaleg et al. 2006; Cloutier et al. 2009, 2012a; Deng et al. 2010, 2011) distributed across the 15 linkage groups of flax (Cloutier et al. 2012b). The amplification products were resolved on an ABI 3130xl Genetic analyzer (Applied Biosystems, Foster City, CA, USA). Output files were analyzed by GeneScan (Applied Biosystems) and subsequently imported into Genographer. Fragment sizes were estimated using GeneScan ROX-500 (Applied Biosystems) and MapMarker^®^ 1000 (BioVentures Inc., Murfreesboro, TN) internal size standards. The genotype of each locus was encoded based on its allele size in bp or as a null allele for dominant markers.

The flax core collection was assessed with 259 mapped neutral SSR loci which indicated that all accessions were organized into two major groups (G1 and G3) and one admixed group (G2) with a weak population structure (*F*_ST_ = 0.09) (Soto-Cerda et al. 2013). G1 included 90 % of the fiber flax accessions mostly from Western Europe and linseed accessions from South Asia and South America, while G3 included accessions from North America and Eastern Europe and was mostly oil type. A relatively fast genome-wide LD decay of ~1 cM (*r* ^2^ = 0.1) was estimated (Soto-Cerda et al. 2013).

Phenotypic data were collected from 390 accessions including 381 accessions selected by Diederichsen et al. (2013) and nine accessions of relevance to recent Canadian flax breeding programs. The 390 accessions were evaluated during 3 years (2009, 2010 and 2011) in Morden, Manitoba (MB) and at the Kernen Farm located near Saskatoon, Saskatchewan (SK), Canada, which represent two mega-environments where most of the linseed is produced in Western Canada (http://www.canadagrainscouncil.ca/). A type 2 modified augmented design (MAD) (Lin and Poushinsky 1985) was used to phenotype oil content and FA composition traits. Main plots (2 m long, 2 m wide with 20 cm row spacing) were arranged in grids of ten rows and ten columns. Each main plot was divided into five parallel subplots of two rows each with a plot control (CDC Bethune replicated 100 times) located at the center. Additional subplot controls (Hanley and Macbeth) were assigned to five randomly selected main plots. The 4-m^2^ plots were harvested, threshed and cleaned. Seeds of each plot were subsampled for oil content and FA composition analyses.

### Oil content and FA composition analyses

OIL was measured by nuclear magnetic resonance calibrated against the FOSFA (Federation of Oils, Seeds and Fats Associations Limited) extraction method. Methyl esters of FA were prepared according to the American Oil Chemists’ Society (AOCS) (http://www.aocs.org/Methods/index.) Official Method Ce 2-66 (09) and FA composition was determined by capillary gas chromatography (GC), following the AOCS Official Method Ce 1e-91. IOD, a measure of the saturation level of lipids, was calculated from the GC-derived FA composition, following the AOCS Method Cd 1c-85.

### Statistical analysis

Adjusted data were obtained for each trait as previously described based on the MAD (You et al. 2013). Normality of the adjusted data was tested using the Shapiro–Wilk test (Shapiro and Wilk 1965) and normal probability plots. The adjusted phenotypic values were used to estimate the variance components to determine the effect of year, location, genotype and their interactions on oil content and FA composition using the GLM procedure in SAS 9.1 (SAS Institute 2004) as described in You et al. (2013). As a measurement of the repeatability of the field trials across years within locations, broad sense heritability (*H*) on an entry mean basis for each seed quality trait was estimated as follows: ${H} = {\sigma}_{G}^{2} / \left[ {\sigma}_{G}^{2} + \left( {{\sigma}_{GE}^{2}} / {e} \right) + \left( {{\sigma}_{E}^{2}} / {e} \; {r} \right) \right]$ where ${\sigma}_{G}^{2}$, ${\sigma}_{GE}^{2}$, ${\sigma}_{E}^{2}$, *e* and *r* correspond to the genetic variance, the genetic by environment interaction variance, the residual variance, the number of environments and the replications per environment, respectively. Pearson’s correlation coefficients (*P* < 0.001) were used to express the relationships between seed quality traits.

### Linkage disequilibrium

An LD heat map was constructed using six linkage groups (LGs) and 158 SSR loci (mean = 1 locus/3.5 cM). The six LGs were selected based on their marker density and differences in size from the consensus linkage map of flax (Cloutier et al. 2012b). The heat map was produced with GGT 2.0 (van Berloo 2008) based on pairwise *r* ^2^ estimates for all marker pairs with minor allele frequency (MAF) > 0.05 (Breseghello and Sorrells 2006). Allelic frequencies were calculated in GENALEX v.6.41 (Peakall and Smouse 2006) and MAF < 0.05 were set to “U” (missing data) and excluded from the LD analysis. This heat map verified the relationships between genomic regions harboring significant markers and large blocks of LD. The 95th percentile of the distribution of unlinked markers *r* ^2^ = 0.09 (Soto-Cerda et al. 2013) was used to set the statistical *r* ^2^ value to determine LD that resulted from physical linkage (Breseghello and Sorrells 2006). Markers on different linkage groups were considered unlinked.

### Association mapping

The adjusted phenotypic values of the seed quality traits were used for AM. Five AM models were tested in TASSEL 2.1 (Bradbury et al. 2007) including two general linear models and three mixed linear models (MLMs). The first GLM incorporated the *Q* matrix as the fixed covariate, while the second used PCA (Price et al. 2006). The first MLM incorporated the kinship matrix (*K*) (Yu et al. 2006) as a random effect only, while the second and third used in addition the *Q* matrix and PCA as fixed covariates, respectively. The *Q* matrix was estimated using 259 mapped neutral SSRs (Soto-Cerda et al. 2013). The PCA matrix calculated in TASSEL 2.1 retained the first three components explaining 27 % of the variation. The *K* matrix was constructed on the basis of 448 SSRs using SPAGeDi (Hardy and Vekemans 2002). All negative values between individuals were set to zero (Yu et al. 2006). The most suitable AM model was selected using cumulative probability–probability (P–P) plots which indicate the extent to which the analysis produced more significant results than expected by chance. For the AM analysis, only MAF > 0.05 were retained (Breseghello and Sorrells 2006).

AM analyses for the seed quality traits were carried out for each year and location independently. Correction for multiple testing was performed using the *q*FDR value, which is an extension of the false discovery rate (FDR) method (Benjamini and Hochberg 1995). The *q* values were calculated with the QVALUE R package using the smoother method (Storey and Tibshirani 2003). Markers with *q*FDR < 0.01 in at least 2 years were considered significant within location. Further, markers with *q*FDR < 0.01 in at least four of the six environments were considered consistent associations. For markers significantly associated with a trait, a GLM with all fixed-effect terms was used to estimate the amount of phenotypic variation explained by each marker (*R* ^2^). Allelic effects of the significant marker loci were calculated as the difference between the average phenotypic values of the homozygous alleles with MAF > 0.05. The significant differences between the allele means were estimated by the Kruskal–Wallis non-parametric test (Kruskal and Wallis 1952) and visualized as box plots.

Candidate QTL were delineated using the estimated background LD (95th percentile) for unlinked markers *r* ^2^ = 0.09 (Soto-Cerda et al. 2013) as suggested by Breseghello and Sorrells (2006). Thus, associated markers were considered linked and part of the same candidate QTL if they showed *r* ^2^ > 0.09. Since markers in the same QTL were closely linked and in significant LD, the amount of phenotypic effect explained by the candidate QTL was estimated using the marker within the QTL with the highest *P* value as described above for the significant markers.

### QTL/marker effects and stability

The QTL/marker effects were calculated as described above for the allelic effects. The stability of a candidate QTL and associated markers was estimated using the additive main effect and multiplicative interaction (AMMI) model (Zobel et al. 1988; Gauch 1992) in GenStat 14 (VSN International 2011). Candidate QTL/markers with a first interaction principal component (IPCA1) near zero are more stable, while those QTL/markers with IPCA1 either positive or negative are more unstable. The AMMI’s stability values (ASV) (Purchase 1997) were also calculated using the following formula:

${\text{ASV}} = \sqrt {\frac{{{\text{SSIPCA}}1}}{{{\text{SSIPCA}}2}}({\text{IPCA}}1)^{2} + ({\text{IPCA}}2)^{2} }$, where SSIPCA1 is the sum of squares interaction of the first principal component (PC) analysis and SSIPCA2 is the sum of squares interaction of the second PC analysis. The smaller the ASV value, the more stable the candidate QTL/markers are across environments. The stability of QTL/markers based on their IPCA1 was defined as follows: 0 to ±0.5 highly stable; ±0.51 to ±1 stable; ±1.01 to ±1.5 moderately stable; and higher than ±1.51 unstable. The stability of QTL/markers based on their ASV values was defined as follows: 0–0.5 highly stable; 0.51–1 stable; 1.01–1.5 moderately stable; and higher than 1.51 unstable. The QTL/marker effects estimated were decomposed into PCs via singular value decomposition and the first two PCs were plotted for both QTL/markers and environments to form a QTL main effect and QTL by environment interaction (QQE) biplot (Yan and Tinker 2005) using GenStat 14 (VSN International 2011).

### Frequency of QTL/marker allele in the flax core collection and Canadian cultivars

QTL/marker alleles were defined as alleles of the marker with the largest *P* value from a QTL or alleles of a significantly associated marker not part of a candidate QTL. With the aim of identifying new potentially favorable QTL/marker alleles absent in linseed Canadian cultivars, the observed number of alleles, the number of private alleles and the allelic richness were contrasted for the 30 linseed Canadian cultivars (Online Resource 1) present in the flax core collection with the remaining 377 of diverse origins (Diederichsen et al. 2013; Soto-Cerda et al. 2013). In addition to the QTL, stable associated markers not part of a QTL but that explained at least 1 % of the phenotypic variation were also included. The number of private QTL/marker alleles and QTL/marker allelic richness were corrected for sample size differences and estimated using the rarefaction method implemented in HP-RARE v.1.2 (Kalinowski 2005). This analysis included all alleles, even the rare ones (MAF < 0.05). The frequencies of the most favorable QTL/marker alleles were estimated in GENALEX v.6.41 (Peakall and Smouse 2006) and compared between the flax core collection and the 30 Canadian cultivars across all identified stable QTL/markers. Significant differences between the allele frequencies were ascertained by the Kruskal–Wallis non-parametric test (Kruskal and Wallis 1952).

## Results

### Phenotypic data

All seed quality traits showed significant genotype (G), location (L) and year (Y) effects (*P* < 0.001; Online Resource 2), although G explained a much larger percentage of the phenotypic variation (33.3–90.6 %) than L (1.2–26.5 %) and Y (0.5–7.3 %). Most of the genotype by environment (GE) interactions (G × L, G × Y, L × Y and G × L × Y) were significant and accounted for up to 10 % of the seed quality traits variation. The location means, standard deviations, ranges, *H* and the correlations exhibited by the seed quality traits are summarized in Table 1. In MB, *H* ranged from 0.87 to 0.99, while in SK, it ranged from 0.73 to 0.98, with a lower mean (0.89) than MB (0.95), indicating that the repeatability between years was more consistent in MB than in SK. LIN and IOD were highly correlated at both locations (MB = 0.87, SK = 0.76; *P* < 0.001). Highly significant negative correlations were observed between the other FAs and IOD. Most of the correlations between FAs were significant and negative. OIL was positively correlated with PAL at both locations and with STE and OLE in SK, but negatively correlated with LIO and IOD in SK.

**Table 1:** Mean ± standard deviation, range, broad sense heritability (*H*) and correlation of seven seed quality traits in the flax core collection evaluated in six environments

Trait	Location	Mean ± SD	Min–max	*H*	OIL	PAL	STE	OLE	LIO	LIN	IOD
OIL	MB	41.6 ± 1.9	33.4–49.7	0.87	–						
	SK	43.3 ± 2.3	32.8–52.3	0.87							
PAL	MB	5.7 ± 0.7	3.3–9.2	0.96	0.21*	–					
	SK	5.4 ± 0.6	3.3–8.4	0.90	0.39*						
STE	MB	4.7 ± 1.2	2.3–11.9	0.97	0.0NS	0.35*	–				
	SK	4.0 ± 0.9	2.2–9.1	0.95	0.24*	0.25*					
OLE	MB	23.8 ± 3.7	15.3–43.9	0.93	0.03NS	0.07NS	0.34*	–			
	SK	18.1 ± 2.9	11.7–35.9	0.90	0.22*	0.11*	0.38*				
LIO	MB	13.6 ± 4.5	4.9–69.2	0.99	−0.06NS	−0.12*	−0.18*	−0.30*	–		
	SK	14.6 ± 4.5	6.6–70.0	0.98	−0.20*	−0.02NS	−0.17*	−0.23*			
LIN	MB	52.2 ± 5.3	3.6–65.4	0.96	−0.01NS	−0.19*	−0.36*	−0.54*	−0.58*	–	
	SK	57.9 ± 5.0	4.7–68.0	0.96	−0.05NS	−0.18*	−0.29*	−0.46*	−0.73*		
IOD	MB	180.7 ± 8.4	143.1–200.3	0.95	−0.03NS	−0.38*	−0.63*	−0.78*	−0.14*	0.87*	–
	SK	192.0 ± 8.0	134.4–208.4	0.73	−0.13*	−0.31*	−0.50*	−0.58*	−0.33*	0.76*	

*OIL* Oil content, *PAL* palmitic acid, *STE* stearic acid, *OLE* oleic acid, *LIO* linoleic acid, *LIN* linolenic acid, *IOD* iodine value, *NS* non-significant

* Significant at *P* < 0.001

### Linkage disequilibrium

As shown in Online Resource 3, syntenic *r* ^2^ (estimated LD for the loci on the same LG) was predominant on LGs 3, 8, 12 and 14, while LGs 1 and 10 showed *r* ^2^ close to background level. Blocks of LD among unlinked loci, which can produce false-positive associations, were also identified suggesting that the kinship matrix used in the MLM could be used to control false-positive LDs (Yu et al. 2006).

### AM analysis

The average relative kinship between any two genotypes was 0.023, and 80 % of the pairwise kinship comparisons ranged from 0 to 0.05 (Online Resource 4). As depicted by the cumulative P–P plots (Online Resource 5), numerous spurious associations for all traits were observed with the GLM (*Q*). This model was characterized by an excess of small *P* values indicating spurious associations. On the other hand, the GLM (PCA) overcorrected the majority of the small *P* values with few higher *P* values departing at the very end of the expected distribution. The MLMs (*K*) and (*Q* + *K*) performed similarly for the seven seed quality traits with their observed *P* values deviating the most from the expected ones for OIL, PAL, STE, OLE, LIO and IOD, indicating that inclusion of the *Q* matrix brought little or no improvement to the AM model. Nevertheless, they displayed a better distribution of *P* values for LIN (Online Resource 5). The MLM (PCA + *K*) had the smallest deviation from the expected distribution for all seed quality traits. The three first PCAs in combination with the *K* matrix were sufficient to control the majority of the potential false-positive associations created by population and family structure. As a result, the *P* values generated by the MLM PCA + *K* were retained for posterior analyses.

### QTL contributing to seed quality traits

**Fig. 1:**
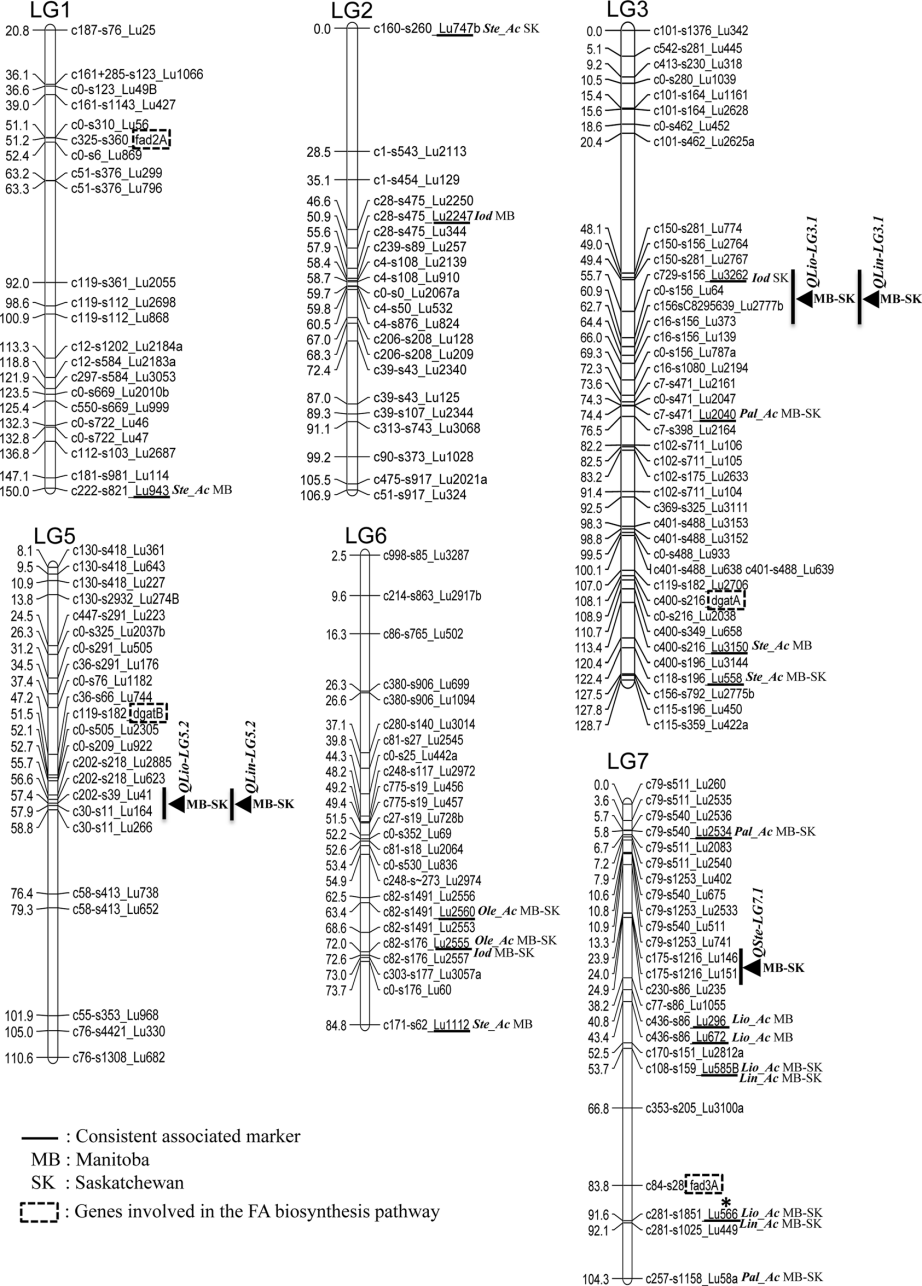

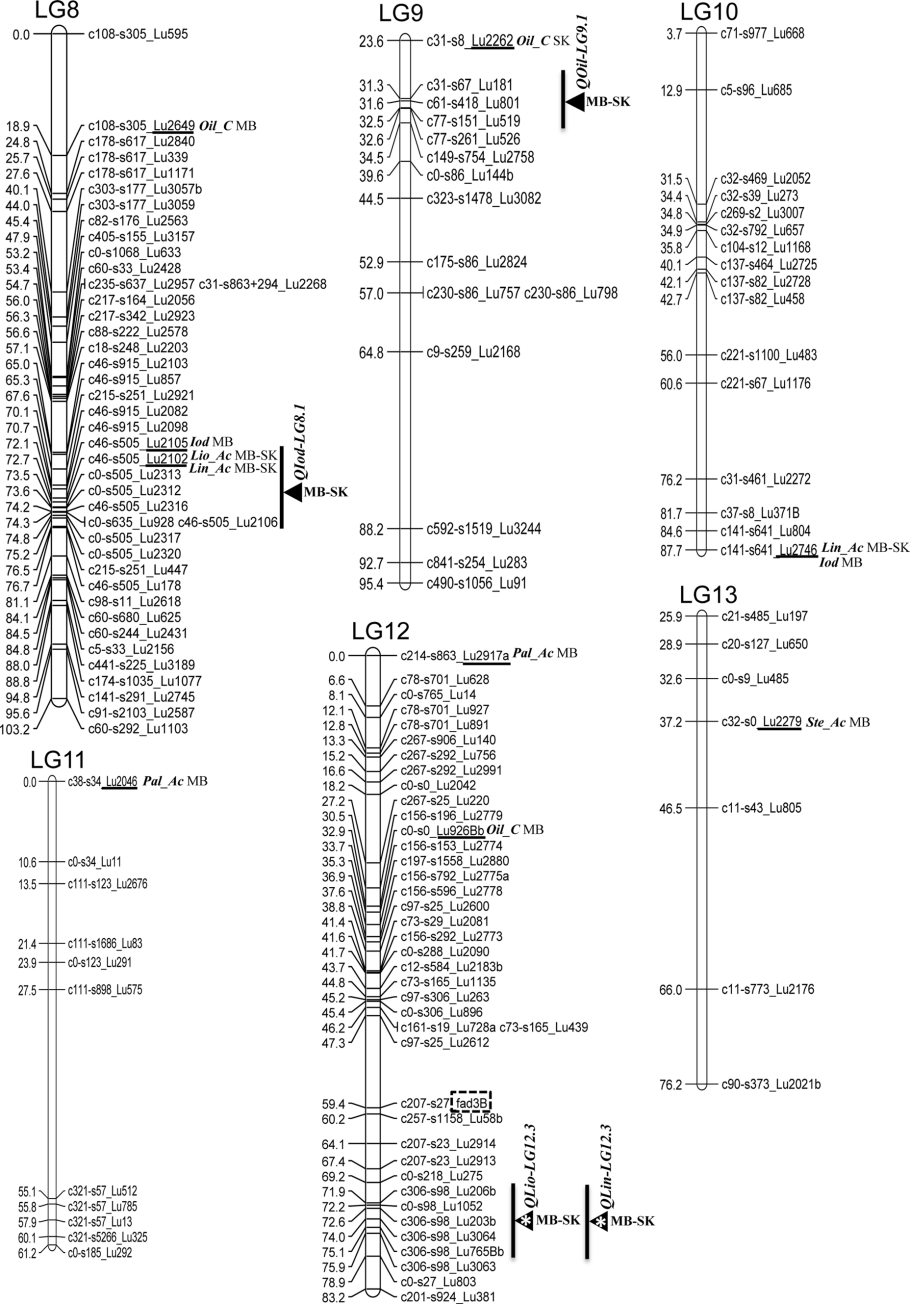
Consensus genetic map of flax (Cloutier et al. 2012b) showing the location of the stable associated markers and candidate QTL for seven seed quality traits in linseed. *Asterisks* indicate QTL previously reported (Cloutier et al. 2011). LGs 4, 14 and 15 are not shown because no stable associations were detected

**Fig. 2:**
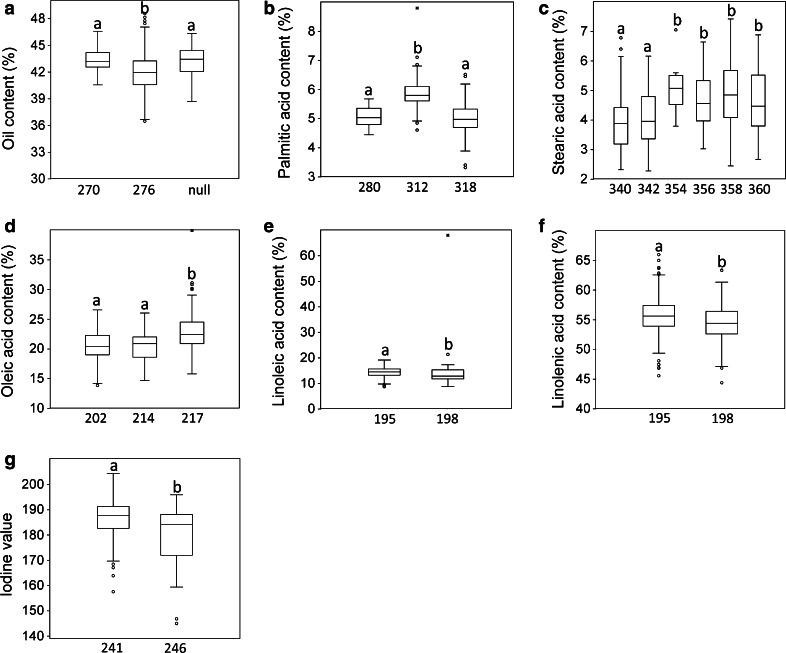
Comparison of allelic effects of seven consistent associated markers with seed quality traits in linseed. **a** Lu181 associated with oil content **b** Lu2534 associated with palmitic acid content **c** Lu146 associated with stearic acid content **d** Lu2555 associated with oleic acid content **e** and **f** Lu3262 associated with linoleic and linolenic acid content **g** Lu2102 associated with iodine value. *Bottom* *values* represent the allele size in base pairs. *Box* *plots* followed by the same letter do not differ statistically according to the Kruskal–Wallis test (*α* = 0.01)

AM was conducted on OIL, PAL, STE, OLE, LIO, LIN and IOD across six environments of the Canadian Prairies. The genomic distribution and number of significant markers, candidate QTL and their phenotypic contribution to seed quality traits are summarized in Fig. 1, Tables 2 and 3 and Online Resource 6. Nine QTL were detected for five seed quality traits. The QTL with the largest effects were *QIod*-*LG8.1, QLin*-*LG5.2* and *QOil*-*LG9.1* for IOD, LIN and OIL, respectively (Table 3). No QTL were detected for PAL and OLE, but marker Lu2046 on LG2 and marker Lu2555 on LG6 explained 8.4 and 3.9 % of the variation, respectively, with one allele contributing significantly to PAL and OLE as described in the next section (Fig. 2b, d). Several QTL and markers co-located within the same chromosomal regions such as those for LIO and LIN on LGs 3, 5 and 12 and LIO, LIN and IOD on LG8 (Fig. 1).

**Table 2:** Summary of significant markers and candidate QTL associated with seven seed quality traits in linseed identified using the MLM (PCA + *K*)

	−Log_10_ (P) threshold	No. of significant markers	% phenotypic variance (*R* ^2^)^a^	No. of candidate QTL	% phenotypic variance (*R* ^2^)^a^
Manitoba (MB)					
Oil content	3.3	7	16.8	1	3.7
Palmitic acid	3.0	4	11.4	0	0
Stearic acid	3.4	10	42.2	1	13.2
Oleic acid	3.6	2	5.5	0	0
Linoleic acid	3.6	15	40.6	3	34.3
Linolenic acid	3.6	12	29.5	3	25.6
Iodine value	3.6	6	12.1	1	5.6
Saskatchewan (SK)					
Oil content	3.5	3	13.8	1	12.8
Palmitic acid	3.1	3	5.3	0	0
Stearic acid	3.2	7	31.9	1	8.2
Oleic acid	3.8	2	6.4	0	0
Linoleic acid	3.5	13	38.1	3	31.8
Linolenic acid	3.5	12	30.2	3	27.0
Iodine value	3.1	5	13.3	1	5.8
Both locations					
Oil content	3.3	2	9.3	1	9.3
Palmitic acid	3.0	2	3.2	0	0
Stearic acid	3.2	3	11.7	1	19.6
Oleic acid	3.7	2	6.2	0	0
Linoleic acid	3.5	13	37.4	3	23.5
Linolenic acid	3.5	12	30.3	3	20.7
Iodine value	3.2	2	7.4	1	6.5

QTL details can be found in Table 3 and Online Resource 6

^a^Total phenotypic variation explained by the associated markers and candidate QTL

**Table 3:** Stable candidate QTL associated with seed quality traits identified at both Manitoba (MB) and Saskatchewan (SK) locations

Trait	Contig–scaffold-marker	Allele size (bp)	LG	Position	−Log_10_ (P)	QTL	Size (cM)	*R* ^2^ (%)	LD (*r* ^2^)^a^	Effect	IPCA1	ASV
OIL	c31-s67_Lu181	270	9	31.34	3.73	*QOil*-*LG9.1*	1.20	7.56	0.27	1.33**	−1.062	2.38
STE	c175-s1216_Lu146	354	7	23.95	6.23	*QSte*-*LG7.1*	0.01	19.68	0.71	1.67**	−0.241	0.40
LIO	c729-s156_Lu3262	217	3	55.74	5.10	*QLio*-*LG3.1*	8.70	6.60	0.24	1.09**	−0.701	1.67
	c30-s11_Lu164	211	5	57.89	3.52	*QLio*-*LG5.2*	0.90	3.31	0.11	0.43*	1.239	2.16
	c306-s98_Lu765Bb	Null	12	75.12	8.40	*QLio*-*LG12.3* ^b^	3.20	13.6	0.93	0.90*	0.489	0.78
LIN	c729-s156_Lu3262	217	3	55.74	5.57	*QLin*-*LG3.1*	8.70	5.33	0.24	1.24**	0.501	1.09
	c202-s39_Lu41	323	5	57.36	5.99	*QLin*-*LG5.2*	0.90	9.31	0.11	1.79**	0.302	1.04
	c306-s98_Lu765Bb	Null	12	75.12	4.86	*QLin*-*LG12.3* ^b^	3.20	6.06	0.93	0.63*	−0.890	1.16
IOD	c46-s505_Lu2102	241	8	72.74	4.23	*QIod*-*LG8.1*	1.60	9.35	0.22	9.31**	0.807	1.05

Significance of the allelic effects tested by Kruskal–Wallis non-parametric test * *P* < 0.01; ** *P* < 0.001

^a^Strength of the physical linkage between markers ranges from 0 (no linkage or no correlation between alleles at different loci) to 1 (total linkage or perfect correlation between alleles at different loci)

^b^Candidate QTL previously reported (Cloutier et al. 2011)

### Allelic effects of stable associations

Some alleles were significantly associated with positive improvements of the traits. For example, the 270 bp allele of Lu181 significantly increased OIL by an average of 1.3 % (*P* < 0.001) across the six environments tested (Fig. 2a). For Lu2534, the 312 bp allele had the largest effect on PAL increasing the value by ~1 % over the average of the other alleles (*P* < 0.001; Fig. 2b). For STE, the 356, 358 and 360 bp alleles of Lu146 had significantly larger effect than the other two alleles (Fig. 2c). An increase of 2.3 % (*P* < 0.001) in OLE was associated with the 217 bp allele of Lu2555 (Fig. 2d). Lu3262 explained ~8 % of the variation for LIO with the 195 bp allele increasing the trait by 0.9 % (Fig 2e). The same allele was also associated with an increase in LIN of 1.3 % (Fig. 2f). A significant positive effect of the 241 bp allele of Lu2102 increased IOD by 9.5 units (Fig. 2g) (*P* < 0.001).

### QTL stability and QTL main effect

The AMMI analysis revealed that four of the nine candidate QTL identified for five seed quality traits were highly stable with IPCA1 values lower than ±0.5 (Table 3). Also, all but three of the candidate QTL were stable or moderately stable with ASV in the range of 0.4–1.16.

**Fig. 3:**
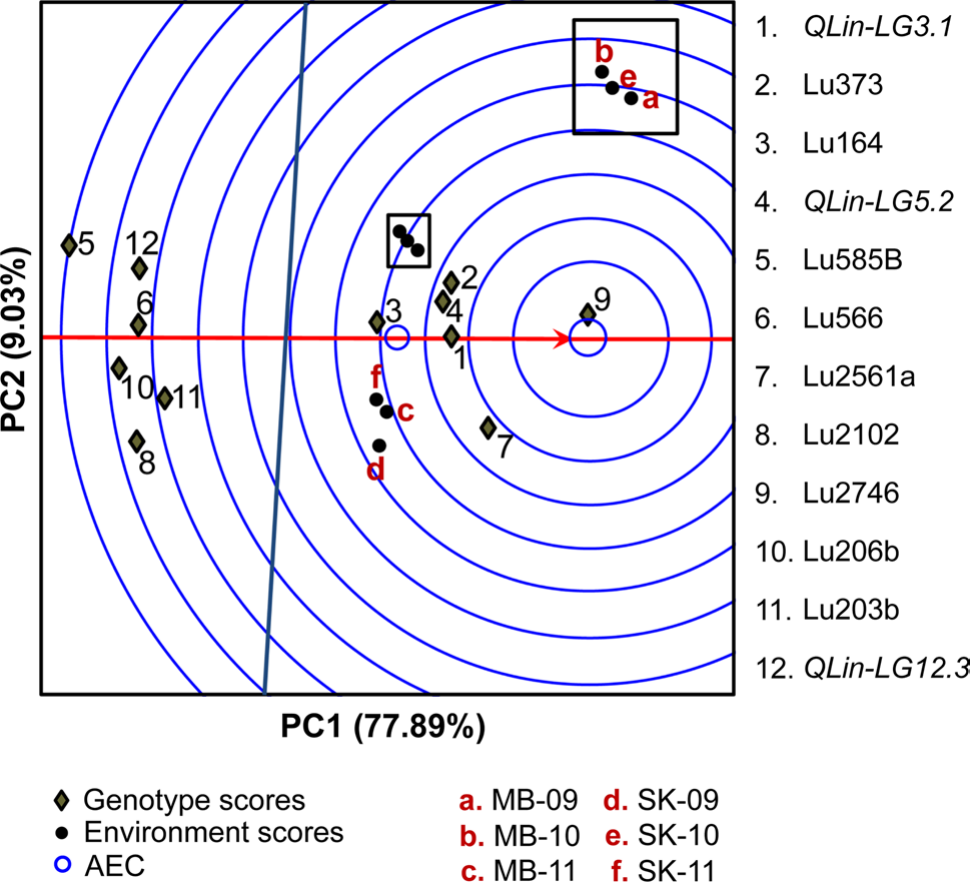
QQE biplot for QTL main effect and QTL stability of linolenic acid content

The QQE biplot displays the average environment defined by the average PC1 and PC2 scores across environments (indicated by an open blue circle) (Fig. 3). The arrow passing through the biplot origin is called the AEC abscissa and points toward increasing QTL main effect. The AEC ordinate line, perpendicular to the abscissa and also passing through the biplot origin, indicates stability/instability. Highly unstable QTL have longer projections on the AEC ordinate irrespective of their direction. The LIN-related candidate QTL/markers were highly stable because most of them landed on or very close to the AEC abscissa (Fig. 3). The intersection of the two axes defines the average QTL/marker main effect, and, as such, Lu203b, Lu2102, Lu206b, Lu566, *QLinLG12.3* and Lu585B had effects below average, while Lu2746, Lu2561a, *QLin*-*LG3.1*, *QLin*-*LG5.2*, Lu373 and Lu164 had the largest main effects on LIN across environments. In general, the QTL main effects showed by the QQE biplot were in agreement with the estimated phenotypic effect (Table 3, Online Resource 6).

### Frequency of QTL/marker allele in the flax core collection and Canadian cultivars

Nine QTL/markers and 16 associated markers not part of a QTL but that explained at least 1 % of the phenotypic variation were included in the analyses, totaling 25 QTL/markers, where some of them were associated with more than one trait (Table 3, Online Resource 6). 43 QTL/marker alleles were present in the 30 lines representing the Canadian cultivars (Online Resource 1) and 102 were present in the remaining 377 lines of the core collection, while the observed number of private QTL/marker alleles, which are alleles exclusively present in a group and absent in the other, was 1 and 77, respectively. After adjusting for sample size differences, the QTL/marker allelic richness was estimated at 43 and 71 in the Canadian cultivars and the core collection respectively, while the number of private QTL/marker alleles was 4 and 32, respectively. In the core collection, 65 of the observed QTL/marker alleles were rare (MAF < 0.05), whereas in the Canadian cultivars only 2 fell in this category (data not shown).

The frequencies of the favorable QTL/marker alleles, i.e., alleles associated with increased OIL and LIN, were not statistically different between the core collection and the Canadian cultivars for the seven seed quality traits (Kruskal–Wallis *P* = 0.437; Online Resource 7). Nevertheless, for most favorable QTL alleles, the Canadian cultivars had higher frequencies, indicating that Canadian flax breeders have been successful in pyramiding the best QTL alleles for seed quality traits. Five favorable alleles were absent in the Canadian cultivars, but were also low in frequency in the core collection (Online Resource 7).

## Discussion

Linseed oil and its FA profile define to a large extent its market end use and value. Genetic progress can be accelerated once genetic diversity for the traits of interest and QTL architecture knowledge are available to breeders. In the present study, we described the application of AM using a core collection of 390 *L. usitatissimum* accessions for the identification of QTL underlying seed quality traits. This study establishes a framework to understand the quantitative nature of OIL and FA composition in linseed.

### Phenotypic analysis

Significant GE interaction was observed for all seven seed quality traits, suggesting genotypic sensitivities to differences in environmental conditions (Online Resource 2). In linseed, OIL and FA composition are affected by temperature during plant development (Casa et al. 1999; Fofana et al. 2006). Differences in planting dates and soil moisture can also affect OIL and FA composition in oil crops (van der Merwe et al. 2013). Fofana et al. (2006) showed that warmer and drier environmental conditions resulted in approximately 5 % lower LIN compared to OLE and suggested that the fatty acid desaturase FAD2, which converts OLE into LIO, was more sensitive to environmental variations and therefore rate limiting. QTL for FA composition had already been linked to the FAD2 enzymes in flax (Cloutier et al. 2011). Our results are in line with this report where OLE was 5.7 % higher in MB than in SK but LIN was higher in SK by the same percentage. Historical meteorological data (30 year period) indicate that the MB location is warmer than the SK location, particularly during the growing season in 2010 and 2011 (Agriculture and Agri-Food Canada; http://climate.weather.gc.ca/advanceSearch/searchHistoricData_e.html).

Broad sense heritability (*H*) estimates were moderate to high with the phenotypic means and ranges reflecting the broad variation of the core collection and also indicating that a large proportion of the phenotypic variation was genetic. Genetic gain could be achieved through phenotypic selection; however, the correlations among seed quality traits exhibited complex relationships. The development of linseed cultivars with specific FA profiles could be better achieved through MAS for which a clear understanding of the genetic architecture of seed quality traits is needed.

### AM analysis

The advantages of AM in identifying QTL for multiple traits in a single diverse population have been outlined (Gupta et al. 2005; Myles et al. 2009; Rafalski 2010). However, this approach sometimes suffers from an inflation of false positives due to population structure (Pritchard et al. 2000) and familial relatedness (Yu et al. 2006). Several linear and mixed models have been proposed to correct for the effect of both confounding factors (Pritchard et al. 2000; Price et al. 2006; Yu et al. 2006). In general, when population and family structures are present, the MLM is superior to the GLM (Myles et al. 2009), but in many cases, the best fitting model will depend on the dynamics of the association panel chosen. The *K* matrix can account for subtle population structure caused by familial relatedness, while the *Q* and PCA matrices control factors such as growth habit, market classes, geography, etc. PCA axes of variation have been shown to better adjust for allele frequency differences between subpopulations (Price et al. 2006; Ma and Amos 2012). In our previous study, one of the two major STRUCTURE sub-groups clustered more than 90 % of the fiber flax accessions, indicating that the inferred *Q* matrix mostly accounted for plant morphotype differences (Soto-Cerda et al. 2013) and, hence, geographic differences present in the flax core collection might not be properly interpreted by the *Q* matrix fitted (Price et al. 2006). For all seven seed quality traits studied herein, the PCA + *K* model provided the best adherence to the expected cumulative distribution of *P* values (Online Resource 5), being superior to the *K* and *Q* + *K* models. This suggests that, in the case of linseed, the PCA matrix can better correct for population stratification, which turns out to also be computationally advantageous even with thousands of markers (Price et al. 2006).

### Fatty acid QTL

Seed oils are composed primarily of triacylglycerols (TAGs), which are glycerol esters of FAs (Rao et al. 2008). The primary FAs in the TAGs of oilseed crops are 16–18 carbons in length and contain 0–3 double bonds where PAL, STE, OLE, LIO and LIN predominate (Rao et al. 2008). Only three FA-related QTL have been identified to date in flax: two co-located QTL, each associated with LIO, LIN and IOD, and one affecting PAL (Cloutier et al. 2011). In the present study, we validated one of them, i.e., the co-located *QLio*-*LG12.3* and *QLin*-*LG12.3* (Fig. 1; Table 3) located in the block of LD on LG12 (Online Resource 3). Several markers and candidate QTL mapped close to genes involved in the FA biosynthesis pathway. Marker Lu3150 (LG3), associated with STE, mapped 5.3 cM from the acyl-CoA:diacylglycerol acyltransferase A (*dgatA*) gene (Fig. 1). Cloutier et al. (2011) mapped the gene using the microsatellite markers present in the upstream region of the *dgat1* gene which was characterized from a bacterial artificial chromosome (BAC) clone. Highly significant associations between *DGTA1*-*2* and OLE and OIL have been reported in maize (Chai et al. 2012). A direct role for DGAT in STE is not obvious because DGAT-A and -B exert their main control in the final steps of oil assembly and are hypothesized to be a determining factor of OIL in higher plants (Weselake 2005). The associations with STE may be caused by the LD between the *dgatA* gene and the putative causative gene, a causal effect which could be resolved with a higher marker density. On the other hand, some of the oil assembly enzymes have been shown to have a preference for certain FAs (Sørensen et al. 2005). Such a selective mechanism could explain their indirect influence on the FA composition because most of the FAs will be assembled in TAGs.

Marker Lu566 (LG7) associated with LIO and LIN co-localized to the same region as the *fad3A* gene, overlapping with the previously published QTL *QLio.crc*-*LG7* and *QLin.crc*-*LG7* (Cloutier et al. 2011), thus being a major candidate gene for the control of LIN. Three *fad3* genes have been identified in the flax genome: *fad3a* and *fad3b* from cultivar Normandy (Vrinten et al. 2005) and more recently *fad3c* (Banik et al. 2011). FAD3A and FAD3B are major enzymes controlling LIN content in linseed (Vrinten et al. 2005); they were mapped in a bi-parental population (Cloutier et al. 2011) and recently integrated into the consensus map of flax (Cloutier et al. 2012b). In linseed, DGATA has an enhanced specificity for α-18:3-CoA (Sørensen et al. 2005; Rao et al. 2008); hence, higher LIN could translate to higher OIL in favorable environments such as SK where LIN was 5.7 % higher and OIL was 1.7 % higher than at the MB location (Table 1).

The genetic architecture of the traits provides some insights into the detection of more QTL for FA composition as compared to OIL. Variations in FA composition are mainly determined by a small number of major genes including fatty acid elongases and desaturases, while the number of genes potentially involved in OIL is expected to be greater and also more sensitive to environmental variations (Honsdorf et al. 2010). The marker density also likely played a role. The 460 SSRs represent less than one-third of the 1,500 estimated minimum markers necessary to tag all QTL, indicating that potentially many QTL remained undetected. Likewise, the flax morphotype, i.e., oilseed and fiber flax, could negatively impact on the number of significant associations. When alleles segregate across multiple subpopulations, MLMs are more powerful, but when they segregate in only one or a subset of the subpopulations or, when different alleles are present in the subpopulations, MLMs will fail to detect the associations entirely (Zhao et al. 2011). We cannot discard the potential effect of the fiber morphotype on seed quality traits associations because it is likely that the favorable alleles associated with these traits do not segregate homogeneously across sub-groups, or they could even be totally absent in the fiber accessions which have not been selected for these traits, consequently underpowering the AM results. AM analysis conducted separately for the fiber and oilseed accessions could provide further insights in this regard.

The phenotypic correlations between traits were consistently reflected in the identification of common markers and candidate QTL (Fig. 1) as reported in other QTL studies (Bachlava et al. 2009; Honsdorf et al. 2010; Cloutier et al. 2011; Hamdan et al. 2012; Li et al. 2012). For example, the stable QTL defined by markers Lu2102 and Lu928 on LG8 (Fig. 1) was associated not only with IOD, but also with LIN which were positively correlated. Another candidate QTL between markers Lu206b and Lu765Bb on LG12 (Fig. 1), associated with both LIO and LIN, overlapped with the previously reported QTL *QLio.crc*-*LG16* and *QLin.crc*-*LG16* having significant negative correlations (Cloutier et al. 2011). Negative correlation between LIO and LIN has been observed in *Brassica* *napus* (Honsdorf et al. 2010) and common QTL affecting several FAs have also been reported in soybean (Bachlava et al. 2009; Xie et al. 2012) and safflower (Hamdan et al. 2012).

### Marker/QTL effects and QTL stability

To maximize the initial impact of MAS in crops with a lack of molecular tools, such as linseed, the associated markers should be closely linked to the QTL and the mapped QTL should ideally have large effect and high stability. For example, the two QTL associated with LIO and LIN reported by Cloutier et al. (2011) were located in a confidence interval of 11.6 cM. In our study, we narrowed down those QTL to 3.2 cM and showed their high stability and high LD (Table 3). Improvement in linkage tightness translates into recombination probability reduction, thus creating better markers for MAS. Nevertheless, because large effect and highly stable QTL will be first fixed in breeding programs, large effect and environment-specific QTL should also be targeted by breeders. For example, *QOil*-*LG9.1*increased OIL by 1.3 % but exhibited higher instability than the other QTL (Table 3). Although our statistical threshold for linked LD was 0.1 which could be considered weak for effective MAS, seven of the identified candidate QTL showed moderate to high LD in the range of 0.22–0.93. However, the phenotypic variation explained by the same QTL differed between studies. In Cloutier et al. (2011), the QTL associated with LIO and LIN explained 20 % each of the variation, higher than the 13.6 and 6.1 % reported in the present study. Many AM studies in humans have reported low *R* ^2^ values, labeling the remaining unexplained variation as the missing heritability (Myles et al. 2009). In *Brassica napus*, 57 significant markers explained up to 18 % of the phenotypic variation for OIL (Zou et al. 2010), while in maize 26 loci explained up to 83 % (Li et al. 2013). There are several reasons for this. First, insufficient marker coverage where the causal polymorphism is not in perfect LD with the genotyped markers affects the detection power of AM leaving unexplained a higher percentage of the variation (Myles et al. 2009). Second, rare alleles with large effects remained undetected because they were excluded for statistical reasons (Breseghello and Sorrells 2006; Rafalski 2010). Third, traits controlled by a large number of genes/QTL, each with small individual effects, may escape statistical detection (Manolio et al. 2009). Fourth, variation resulting from epistatic interactions between genes might also go undiscovered because epistasis can only be investigated practically in a sequential scan of major common loci (Storey et al. 2005). Finally, epigenetic variations are emerging as a major cause of the missing heritability (Rakyan et al. 2011). Epigenome-wide association studies are likely going to shed some light on the specific epigenetic mechanisms at play in phenotypic variation (Rakyan et al. 2011), and most interestingly their environmental and trans-generational stabilities. Bi-parental mapping has the power to detect the effects of rare alleles (Gupta et al. 2005). As such, high *R* ^2^ values reported by Cloutier et al. (2011) using a bi-parental cross of high LIN with low LIN, providing an extreme range of FA profiles, likely correspond to the mutant parental line major fatty acid desaturase rare alleles of large effect, while in AM the smaller *R* ^2^ values could correspond to common variants of small effects from the same locus. Allele frequency differences for the same underlying locus between bi-parental populations and AM panels affect the explained phenotypic variation (Stich et al. 2008). The maximum proportion of the variance explained by a marker is observed for allele frequencies of 0.5, as expected in bi-parental populations such as recombinant inbred lines or F_1_-derived doubled haploids. For an AM panel, the allele frequencies are expected to be considerably different from 0.5, especially when multi-allelic markers such as SSRs are used (Stich et al. 2008). As a consequence, the proportion of the variance explained by a marker is notably lower despite the same underlying allelic effect (Stich et al. 2008). In our study, the majority of the associated markers and candidate QTL explained <5 % of the phenotypic variation. Nevertheless, some candidate QTL explained up to 19 % of the phenotypic variation, and major QTL for OIL (8 %), STE (19.6 %), LIO (6.6 %) and LIN (9.3 %) were stable, making them suitable for MAS (Table 3; Fig. 3).

### Frequency of QTL/marker allele in the flax core collection and Canadian cultivars

Several reports indicate that Canadian linseed cultivars have been developed from a narrow genetic base (Fu et al. 2002, 2003; Cloutier et al. 2009) which is an impediment to further breeding progress. In the present study, the flax core collection showed abundant QTL allelic diversity with approximately eight times more unique (private) alleles than the Canadian cultivar subgroup. However, the majority of these novel QTL alleles were rare, limiting their exploitation in AM, hence requiring different strategies for their efficient utilization. Among these potential strategies, optimal bi-parental mapping populations could be designed using the comprehensive phenotypic and genetic characterization of the flax core collection. In addition, the joint use of linkage mapping and association models through the design of multiparent advanced generation intercross (MAGIC) or nested association mapping (NAM) populations can overcome the population structure issue (Rafalski 2010). These populations are advantageous from the point of view of increasing the frequency of rare alleles and balancing the overall allele frequencies, although the strong kinship relationships could be an impediment. However, the high kinship relationships among genotypes could be mitigated by MLM and exploited through genomic selection, a strategy complementary to AM which uses genome-wide marker information to model phenotypic traits and obtain estimated breeding values (Meuwissen et al. 2001).

### Final remarks

The current study represents the first AM analysis in linseed. We identified nine consistent QTL across six environments for seed quality traits and several stable markers providing a basis for further AM and fine mapping efforts aiming to understand the genetic architecture of seed quality traits in linseed. Although this study was somewhat limited with respect to marker density, novel QTL were mapped and several previously reported were validated. To realize the full potential of AM and of the flax core collection, whole genome re-sequencing of the entire core collection is under way to saturate the genetic map with hundreds of thousands of single nucleotide polymorphism markers. Validation of candidate QTL in bi-parental populations will guide the development of marketable linseed cultivars using MAS.

## Electronic supplementary material

Below is the link to the electronic supplementary material.

Supplementary material 1 (DOCX 1394 kb)

## References

[CR1] Bachlava E, Dewey RE, Burton JW, Cardinal AJ (2009). Mapping candidate genes for oleate biosynthesis and their association with unsaturated fatty acid seed content in soybean. Mol Breed.

[CR2] Banik M, Duguid S, Cloutier S (2011). Transcript profiling and gene characterization of three fatty acid desaturase genes in high, moderate, and low linolenic acid genotypes of flax (*Linum usitatissimum* L.) and their role in linolenic acid accumulation. Genome.

[CR3] Benjamini Y, Hochberg Y (1995). Controlling the false discovery rate: a practical and powerful approach to multiple testing. J R Statist Soc B.

[CR4] Bradbury PJ, Zhang Z, Kroon DE, Casstevens TM, Ramdoss Y, Buckler ES (2007). TASSEL: software for association mapping of complex traits in diverse samples. Bioinformatics.

[CR5] Breseghello F, Sorrells M (2006). Association mapping of kernel size and milling quality in wheat (*Triticum**aestivum* L.) cultivars. Genetics.

[CR6] Casa R, Russell G, Lo Cascio B, Rossini F (1999). Environmental effects on linseed (*Linum usitatissimum* L.) yield and growth of flax at different stand densities. Eur J Agron.

[CR7] Chai Y, Hao X, Yang X, Allen WB, Li J, Yan J, Shen B, Li J (2012). Validation of *DGAT1*-*2* polymorphisms associated with oil content and development of functional markers for molecular breeding of high-oil maize. Mol Breed.

[CR8] Chung J, Babka HL, Graef GL, Staswick PE, Lee DJ, Cregan PB, Shoemaker RC, Specht JE (2003). The seed protein, oil, and yield QTL on soybean linkage group I. Crop Sci.

[CR9] Cloutier S, Niu Z, Datla R, Duguid S (2009). Development and analysis of EST-SSRs for flax (*Linum usitatissimum* L.). Theor Appl Genet.

[CR10] Cloutier S, Ragupathy R, Niu Z, Duguid S (2011). SSR-based linkage map of flax (*Linum usitatissimum* L.) and mapping of QTLs underlying fatty acid composition traits. Mol Breed.

[CR11] Cloutier S, Miranda E, Ward K, Radovanovic N, Reimer E, Walichnowski A, Datla R, Rowland G, Duguid S, Ragupathy R (2012). Simple sequence repeat marker development from bacterial artificial chromosome end sequences and expressed sequence tags of flax (*Linum usitatissimum* L.). Theor Appl Genet.

[CR12] Cloutier S, Ragupathy R, Miranda E, Radovanovic N, Reimer E, Walichnowski A, Ward K, Rowland G, Duguid S, Banik M (2012). Integrated consensus genetic and physical maps of flax (*Linum usitatissimum* L.). Theor Appl Genet.

[CR13] Cook JP, McMullen MD, Holland JB, Tian F, Bradbury P, Ross-Ibarra J, Buckler ES, Flint-Garcia SA (2012). Genetic architecture of maize kernel composition in the nested association mapping and inbred association panels. Plant Physiol.

[CR14] Cullis CA, Kole C (2007). Flax. Genome mapping and molecular breeding in plants.

[CR15] Deng X, Long S, He D, Li X, Wang Y, Liu J, Chen H (2010). Development and characterization of polymorphic microsatellite markers in *Linum usitatissimum*. J Plant Res.

[CR16] Deng X, Long S, He D, Li X, Wang Y, Hao D, Qiu C, Chen X (2011). Isolation and characterization of polymorphic microsatellite markers from flax (*Linum usitatissimum* L.). Afr J Biotechnol.

[CR17] Diederichsen A, Kusters PM, Kessler D, Bainas Z, Gugel RK (2013). Assembling a core collection from the flax world collection maintained by Plant Gene Resources of Canada. Genet Resour Crop Evol.

[CR18] Duguid SD, Vollmann J, Rajcan I (2009). Flax. Oil crops, handbook of plant breeding 4.

[CR19] Ersoz ES, Yu J, Buckler ES, Kriz A, Larkins B (2009). Applications of linkage disequilibrium and association mapping in maize. Molecular genetic approaches to maize improvement.

[CR20] FAOSTAT (2013) Production of crops: linseed: area harvested and production (tonnes). Available at http://faostat3.fao.org/home/index.html. Accessed March 2013

[CR21] Fofana B, Duguid S, Cloutier S (2004). Cloning of fatty acid biosynthetic genes *β*-ketoacyl CoA synthase, fatty acid elongase, stearoyl-ACP desaturase and fatty acid desaturase and analysis of expression in the early developmental stages of flax (*Linum usitatissimum* L.) seeds. Plant Sci.

[CR22] Fofana B, Cloutier S, Duguid S, Ching J, Rampitsch C (2006). Gene expression of stearoyl-ACP desaturase and Δ12 fatty acid desaturase 2 is modulated during seed development of flax (*Linum usitatissimum*). Lipids.

[CR23] Friedt W, Bickert C, Schaub H (1995). In vitro breeding of highlinolenic, doubled haploid lines of linseed (Linum usitatissimum L.) via androgenesis. Plant Breed.

[CR24] Fu YB, Diederichsen A, Richards KW, Peterson G (2002). Genetic diversity within a range of cultivars and landraces of flax (*Linum usitatissimum* L) as revealed by RAPDs. Genet Resour Crop Evol.

[CR25] Fu YB, Rowland GG, Duguid SD, Richards K (2003). RAPD analysis of 54 North American flax cultivars. Crop Sci.

[CR26] Gauch HG, Kang MS, Gauch HG (1992). AMMI analysis of yield trials. Genotype-by-environment interaction.

[CR27] Goldman IL, Rocheford TR, Dudley JW (1994). Molecular markers associated with maize kernel oil concentration in an Illinois high protein × Illinois low protein cross. Crop Sci.

[CR28] Green AG (1986). A mutant genotype of flax (*Linum usitatissimum* L.) containing very low levels of linolenic acid in its seed oil. Can J Plant Sci.

[CR29] Green AG, Marshall AR (1984). Isolation of induced mutants in linseed (*Linum usitatissimum* L.) having reduced linolenic acid content. Euphytica.

[CR30] Green AG, Chen Y, Singh SP, Dribnenki JCP, Kole C, Hall TC (2008). Flax. Compendium of transgenic crop plants: transgenic oilseed crops.

[CR31] Gupta PK, Rustgi S, Kulwal PL (2005). Linkage disequilibrium and association studies in higher plants: present status and future prospects. Plant Mol Biol.

[CR32] Hamdan YAS, Garcia-Moreno MJ, Fernandez-Martinez JM, Velasco L, Perez-Vich B (2012). Mapping of major and modifying genes for high oleic acid content in safflower. Mol Breed.

[CR33] Hardy OJ, Vekemans X (2002). SPAGeDi: a versatile computer program to analyse spatial genetic structure at the individual or population levels. Mol Ecol Notes.

[CR34] Harris DR (1997) The spread of neolithic agriculture from the Levant to western-central Asia. In: Damania AB, Valkoun J, Willcox G, Qualset CO (eds) The origin of agriculture and crop domestication. Proceedings of Harlan Symposium, ICARDA, Aleppo, Syria, 10–14 May, pp 65–82

[CR35] Honsdorf N, Becker HC, Ecke W (2010). Association mapping for phenological, morphological, and quality traits in canola quality winter rapeseed (*Brassica napus* L.). Genome.

[CR36] Hu X, Sullivan-Gilbert M, Gupta M, Thompson SA (2006). Mapping of the loci controlling oleic and linolenic acid contents and development of *fad2* and *fad3* allele-specific markers in canola (*Brassica napus* L.). Theor Appl Genet.

[CR37] Kalinowski ST (2005). HP-RARE 1.0: a computer program for performing rarefaction on measures of allelic richness. Mol Ecol Notes.

[CR38] Kenaschuk EO (2005) High linolenic acid flax. US patent 6870077 issued on March 22, 2005

[CR39] Khadake RM, Ranjekar PK, Harsulkar AM (2009). Cloning of a novel omega-6 desaturase from flax (*Linum usitatissimum*) and its functional analysis in *Saccharomyces cerevisiae*. Mol Biotechnol.

[CR40] Kruskal WH, Wallis WA (1952). Use of ranks in one-criterion variance analysis. J Am Stat Assoc.

[CR41] Kumar S, You FM, Cloutier S (2012). Genome wide SNP discovery in flax through next generation sequencing of reduced representation libraries. BMC Genom.

[CR42] Li Y, Smulders MJM, Chang R, Qiu L (2011). Genetic diversity and association mapping in a collection of selected Chinese soybean accessions based on SSR marker analysis. Conserv Genet.

[CR43] Li X, Yan W, Agrama H, Jia L, Jackson A, Moldenhauer K, Yeater K, McClung A, Wu D (2012). Unraveling the complex trait of harvest index with association mapping in rice (*Oryza sativa* L.). PLoS One.

[CR44] Li H, Peng Z, Yang X, Wang W, Fu J, Wang J, Han Y, Chai Y, Guo T, Yang N, Liu J, Warburton ML, Cheng Y, Hao X, Zhang P, Zhao J, Liu Y, Wang G, Li J, Yan J (2013). Genome-wide association study dissects the genetic architecture of oil biosynthesis in maize kernels. Nat Genet.

[CR45] Lin CS, Poushinsky G (1985). A modified augmented design (type 2) for rectangular plots. Can J Plant Sci.

[CR46] Ma J, Amos C (2012). Principal components analysis of population admixture. PLoS One.

[CR47] Manolio TA, Collins FS, Cox NJ, Goldstein DB, Hindorff LA, Hunter DJ, McCarthy MI, Ramos EM, Cardon LR, Chakravarti A, Cho JH, Guttmacher AE, Kong A, Kruglyak L, Mardis E, Rotimi CN, Slatkin M, Valle D, Whittemore AS, Boehnke M, Clark AG, Eichler EE, Gibson G, Haines JL, Mackay TF, McCarroll SA, Visscher PM (2009). Finding the missing heritability of complex diseases. Nature.

[CR48] Meuwissen TH, Hayes BJ, Goddard ME (2001). Prediction of total genetic value using genome-wide dense marker maps. Genetics.

[CR49] Myles S, Peiffer J, Brown PJ, Ersoz ES, Zhang Z, Costich DE, Buckler ES (2009) Association mapping: critical considerations shift from genotyping to experimental design. Plant Cell 21:2194–2202 10.1105/tpc.109.068437 PMC2751942 19654263

[CR50] Peakall R, Smouse PE (2006). GENALEX 6: genetic analysis in excel. Population genetic software for teaching and research. Mol Ecol Notes.

[CR51] Price AL, Patterson NJ, Plenge RM, Weinblatt ME, Shadick NA, Reich D (2006). Principal components analysis corrects for stratification in genome-wide association studies. Nat Genet.

[CR52] Pritchard JK, Stephens M, Rosenberg NA, Donnelly P (2000). Association mapping in structured populations. Am J Hum Genet.

[CR53] Purchase JL (1997) Parametric analysis to describe G × E interaction and yield stability in winter wheat. PhD thesis. Department of Agronomy, Faculty of Agriculture, University of the Orange Free State, Bloemfontein, South Africa

[CR54] Qi Z, Wu Q, Han X, Sun Y, Du X, Liu C, Jiang H, Hu G, Chen Q (2011). Soybean oil content QTL mapping and integrating with meta-analysis method for mining genes. Euphytica.

[CR55] Qiu D, Morgan C, Shi J, Long Y, Liu J, Li R, Zhuang X, Wang Y, Tan X, Dietrich E, Weihmann T, Everett C, Vanstraelen S, Beckett P, Fraser F, Trick M, Barnes S, Wilmer J, Schmidt R, Li J, Li D, Meng J, Bancroft I (2006). A comparative linkage map of oilseed rape and its use for QTL analysis of seed oil and erucic acid content. Theor Appl Genet.

[CR56] Rafalski JA (2010). Association genetics in crop improvement. Curr Opin Plant Biol.

[CR57] Ragupathy R, Rathinavelu R, Cloutier S (2011). Physical mapping and BAC-end sequence analysis provide initial insights into the flax (*Linum usitatissimum* L.) genome. BMC Genom.

[CR58] Rakyan VK, Down TA, Balding DJ, Beck S (2011). Epigenome-wide association studies for common human diseases. Nat Rev Genet.

[CR59] Rao S, Abdel-Reheem M, Bhella R, McCraken C, Hildebrand D (2008). Characteristics of high α-linolenic acid accumulation in seed oils. Lipids.

[CR60] Roose-Amsaleg C, Cariou-Pham E, Vautrin D, Tavernier R, Solignac M (2006). Polymorphic microsatellite loci in *Linum usitatissimum*. Mol Ecol Notes.

[CR61] Rowland GG (1991). An EMS-induced low linolenic acid mutant in McGregor flax (*Linum usitatissimum* L.). Can J Plant Sci.

[CR62] Rowland GG, Bhatty RS (1990). Ethyl methanesulfonate induced fatty acid mutations in flax. J Am Oil Chem Soc.

[CR63] SAS Institute (2004) SAS Version 9.1. SAS Institute, Cary

[CR64] Shapiro SS, Wilk MB (1965). An analysis of variance test for normality (complete samples). Biometrika.

[CR65] Simopoulos AP (2000). Human requirement for N-3 polyunsaturated fatty acids. Poult Sci.

[CR66] Smooker AM, Wells R, Morgan C, Beaudoin F, Cho K, Fraser F, Bancroft I (2011). The identification and mapping of candidate genes and QTL involved in the fatty acid desaturation pathway in *Brassica napus*. Theor Appl Genet.

[CR67] Sørensen BM, Furukawa-Stoffer TL, Marshall KS, Page EK, Mir Z, Forster RJ, Weselake RJ (2005). Storage lipid accumulation and acyltransferase action in developing flaxseed. Lipids.

[CR68] Soto-Cerda BJ, Carrasco RJ, Aravena GA, Urbina HA, Navarro CS (2011). Identifying novel polymorphic microsatellites from cultivated flax (*Linum usitatissimum* L.) following data mining. Plant Mol Biol Rep.

[CR69] Soto-Cerda BJ, Urbina Saavedra H, Navarro Navarro C, Mora Ortega P (2011). Characterization of novel genic SSR markers in *Linum usitatissimum* (L.) and their transferability across eleven *Linum* species. Electron J Biotechnol.

[CR70] Soto-Cerda BJ, Diederichsen A, Ragupathy R, Cloutier S (2013). Genetic characterization of a core collection of flax (*Linum usitatissimum* L.) suitable for association mapping studies and evidence of divergent selection between fiber and linseed types. BMC Plant Biol.

[CR71] Stich B, Piepho HP, Schulz B, Melchinger AE (2008). Multi-traits association mapping in sugar beet (*Beta vulgaris* L.). Theor Appl Genet.

[CR72] Storey JD, Tibshirani R (2003). Statistical significance for genomewide studies. Proc Natl Acad Sci USA.

[CR73] Storey JD, Akey JM, Kruglyak L (2005). Multiple locus linkage analysis of genomewide expression in yeast. PLoS Biol.

[CR74] van Berloo R (2008). GGT 2.0: Versatile software for visualization and analysis of genetic data. J Hered.

[CR75] van der Merwe R, Labuschagne MT, Herselman L, Hugo A (2013). Stability of seed oil quality traits in high and mid-oleic acid sunflower hybrids. Euphytica.

[CR76] Vollmann J, Rajcan I, Vollmann J, Rajcan I (2009). Oil crops breeding and genetics. Oil crops, handbook of plant breeding 4.

[CR77] Vrinten P, Hu Z, Munchinsky MA, Rowland G, Qiu X (2005). Two FAD3 desaturase genes control the level of linolenic acid in flax seed. Plant Physiol.

[CR78] VSN International (2011). GenStat for Windows 14th Edition. VSN International, Hemel Hempstead, UK. http://www.GenStat.co.uk

[CR79] Wang ML, Sukumaran S, Barkley NA, Chen Z, Chen CY, Guo B, Pittman RN, Stalker HT, Holbrook CC, Pederson GA, Yu J (2011). Population structure and marker-trait association analysis of the US peanut (*Arachis hypogaea* L.) mini-core collection. Theor Appl Genet.

[CR80] Wang Z, Hobson N, Galindo L, Zhu S, Shi D, McDill J, Yang L, Hawkins S, Neutelings G, Datla R, Lambert G, Galbraith DW, Grassa CJ, Geraldes A, Cronk QC, Cullis C, Dash PK, Kumar PA, Cloutier S, Sharpe AG, Wong GK, Wang J, Deyholos MK (2012) The genome of flax (*Linum usitatissimum*) assembled *de novo* from short shotgun sequence reads. Plant J 72:461–473 10.1111/j.1365-313X.2012.05093.x 22757964

[CR81] Wassom JJ, Wong JC, Martinez E, King JJ, DeBaene J, Hotchkiss JR, Mikkilineni V, Bohn MO, Rocheford TR (2008). QTL associated with maize kernel oil, protein, and starch concentrations; kernel mass; and grain yield in Illinois high oil × B73 backcross-derived lines. Crop Sci.

[CR82] Weselake RJ, Murphy DJ (2005). Storage lipids. Plant lipids: biology, utilization and manipulation.

[CR83] Westcott ND, Muir ND, Muir AD, Westcott ND (2003). Chemical studies on the constituents of Linum sp. Flax, the genus *Linum*.

[CR84] Wilson RF (2012). The role of genomics and biotechnology in achieving global food security for high-oleic vegetable oil. J Oleo Sci.

[CR85] Xie D, Han Y, Zeng Y, Chang W, Teng W, Li W (2012). SSR- and SNP-related QTL underlying linolenic acid and other fatty acid contents in soybean seeds across multiple environments. Mol Breed.

[CR86] Yan W, Tinker NA (2005). A biplot approach for investigating QTL-by-environment patterns. Mol Breed.

[CR87] Yang X, Yan J, Shah T, Warburton ML, Li Q, Li L, Gao Y, Chai Y, Fu Z, Zhou Y, Xu S, Bai G, Meng Y, Zheng Y, Li J (2010). Genetic analysis and characterization of a new maize association mapping panel for quantitative trait loci dissection. Theor Appl Genet.

[CR88] You FM, Duguid SD, Thambugala D, Cloutier S (2013) Statistical analysis and field evaluation of the type 2 modified augmented design in phenotyping of flax germplasms in multiple environments. Australia J Crop Sci 7:1789–1800

[CR89] Yu J, Pressoir G, Briggs W, Vroh Bi I, Yamasaki M, Doebley J, McMullen M, Gaut B, Nielsen D, Holland J, Kresovich S, Buckler E (2006). A unified mixed-model method for association mapping that accounts for multiple levels of relatedness. Nat Genet.

[CR90] Zhao J, Becker HC, Zhang D, Zhang X, Ecke W (2005). Oil content in a European × Chinese rapeseed population: QTL with additive and epistatic effects and their genotype–environment interactions. Crop Sci.

[CR91] Zhao K, Tung CW, Eizenga GC, Wright MH, Ali ML, Price AH, Norton GJ, Islam MR, Reynolds A, Mezey J, McClung AM, Bustamante CD, McCouch SR (2011). Genome-wide association mapping reveals a rich genetic architecture of complex traits in Oriza sativa. Nat Commun.

[CR92] Zobel RW, Wright MG, Gauch HG (1988). Statistical analysis of yield trial. Agron J.

[CR93] Zou J, Jiang C, Cao Z, Li R, Long Y, Chen S, Meng J (2010). Association mapping of seed oil content in *Brassica napus* and comparison with quantitative trait loci identified from linkage mapping. Genome.

