# Supplementary material for: Association mapping of seed quality traits using the Canadian flax (Linum usitatissimum L.) core collection
Source: Theor Appl Genet. 2014 Jan 26;127(4):881–96. doi: 10.1007/s00122-014-2264-4 (PMC3964306; doi:10.1007/s00122-014-2264-4)
Supplement: Supplementary file 1 — Supplementary material 1 (DOCX 1394 kb) [file 122_2014_2264_MOESM1_ESM.docx]

**Association Mapping of Seed Quality Traits using the Canadian Flax (*Linum usitatissimum* L.) Core Collection**

Braulio J. Soto-Cerda^1,2†^, Scott Duguid^3^, Helen Booker^4^, Axel Diederichsen^5^, Sylvie Cloutier^1,2*^

^*^Corresponding author Email: [Sylvie.J. Cloutier@agr.gc.ca](mailto:Sylvie.J.%20Cloutier@agr.gc.ca)

^1^University of Manitoba, Department of Plant Science, 66 Dafoe Road, Winnipeg, MB, R3T 2N2, Canada

^2^Cereal Research Centre, Agriculture and Agri-Food Canada, 195 Dafoe Rd, Winnipeg, MB, R3T 2M9, Canada

^3^Morden Research Station, Agriculture and Agri-Food Canada, Route 100, Morden, MB, R6M 1Y5, Canada

^4^University of Saskatchewan, Crop Development Centre, College of Agriculture and Bioresources, 51 Campus Drive, Saskatoon, SK, S7N 5A8, Canada

^5^Plant Gene Resources of Canada, Agriculture and Agri-Food Canada, 107 Science Place, Saskatoon, SK, S7N 0X2, Canada

^†^Permanent address: Agriaquaculture Nutritional Genomic Center, CGNA, Genomics and Bioinformatics Unit, Km 10 Camino Cajón-Vilcún, INIA, Temuco, Chile

**Online Resources**

**Online Resource 1**

**Table S1** List of Canadian cultivars that are part of the flax core collection

| Canadian number | Name | Donor Institute |
| --- | --- | --- |
| CN18973 | AC Watson | AAFC-Indian Head |
| CN18979 | Flanders | CDC-Saskatoon |
| CN18980 | Somme | CDC-Saskatoon |
| CN18981 | CDC Valour | CDC-Saskatoon |
| CN19003 | AC McDuff | AAFC-Morden |
| CN19004 | AC Emerson | AAFC-Morden |
| CN19005 | AC Linora | AAFC-Indian Head |
| CN19017 | CDC Normandy | CDC-Saskatoon |
| CN33385 | Linott | AAFC-Morden |
| CN33386 | Noralta | AAFC-Morden |
| CN33388 | Redwood 65 | AAFC-Morden |
| CN33389 | Rocket | AAFC-Morden |
| CN33397 | Dufferin | AAFC-Morden |
| CN37286 | McGregor | AAFC-Morden |
| CN52732 | Norlin | FP& I Branch, Seed Division |
| CN100547 | Redwing | AAFC-Regina |
| CN101413 | Vimy | CDC-Saskatoon |
| Linola989 | Linola989 |  |
| CDCGold | CDCGold |  |
| Macbeth | Macbeth |  |
| Shape | Shape |  |
| CDCSorrel | CDCSorrel |  |
| CDCBethune | CDCBethune |  |
| CDCMons | CDCMons |  |
| CrepitamTabor | CrepitamTabor |  |
| Hanley | Hanley |  |
| Lirina | Lirina |  |
| PrairieBlue | PrairieBlue |  |
| PrairieGrande | PrairieGrande |  |
| PrairieThunder | PrairieThunder |  |

AAFC: Agriculture and Agri-Food Canada; CDC: Crop Development Center; FP & I Branch: Food Protection and Inspection

**Online Resource 2**

**Table S2** Analysis of variance for seed quality traits in the flax core collection evaluated in six environments. Mean square values and percentage of the total sum of squares for oil content (OIL), palmitic acid (PAL), stearic acid (STE), oleic acid (OLE), linoleic acid (LIO), linolenic acid (LIN), and iodine value (IOD) are shown

| Source of variation | OIL | %TSS^a^ | PAL | %TSS^a^ | STE | %TSS^a^ | OLE | %TSS^a^ | LIO | %TSS^a^ | LIN | %TSS^a^ | IOD | %TSS^a^ |
| --- | --- | --- | --- | --- | --- | --- | --- | --- | --- | --- | --- | --- | --- | --- |
| Genotype (G) | 28.26* | 53.8 | 2.48* | 76.9 | 7.16* | 72.1 | 62.31* | 33.3 | 116.35* | 90.6 | 148.14* | 55.1 | 342.61* | 39.0 |
| Location (L) | 1166.98* | 5.7 | 28.48* | 2.3 | 259.08* | 6.7 | 19285.56* | 26.5 | 599.85* | 1.2 | 18459.74* | 17.6 | 75216.33* | 22.0 |
| Year (Y) | 748.63* | 7.3 | 14.65* | 2.3 | 66.19* | 3.4 | 2580.76* | 7.1 | 134.25* | 0.5 | 1830.95* | 3.5 | 6879.74* | 4.0 |
| G * L | 2.44* | 4.6 | 0.12* | 3.7 | 0.26* | 2.6 | 3.02* | 1.6 | 0.68* | 0.5 | 3.79* | 1.4 | 31392* | 3.6 |
| G * Y | 2.16* | 8.1 | 0.08* | 5.2 | 0.16* | 3.2 | 2.94* | 3.1 | 1.04* | 1.6 | 3.52* | 2.6 | 28.13* | 6.4 |
| L * Y | 354.34* | 3.5 | 1.27* | 0.2 | 62.97* | 3.3 | 3376.52* | 9.3 | 160.11* | 0.6 | 2904.21* | 5.6 | 11748.27* | 6.9 |
| G * L * Y | 1.73* | 5.7 | 0.07 *n.s.* | 4.1 | 0.13* | 2.4 | 2.32* | 2.3 | 0.53* | 0.8 | 2.88* | 2.0 | 26.61* | 5.7 |

^a^ % TSS = percentage of the total sum of squares

* Significant at *P* < 0.0001; *n.s*. = non-significant

**Online Resource 3**


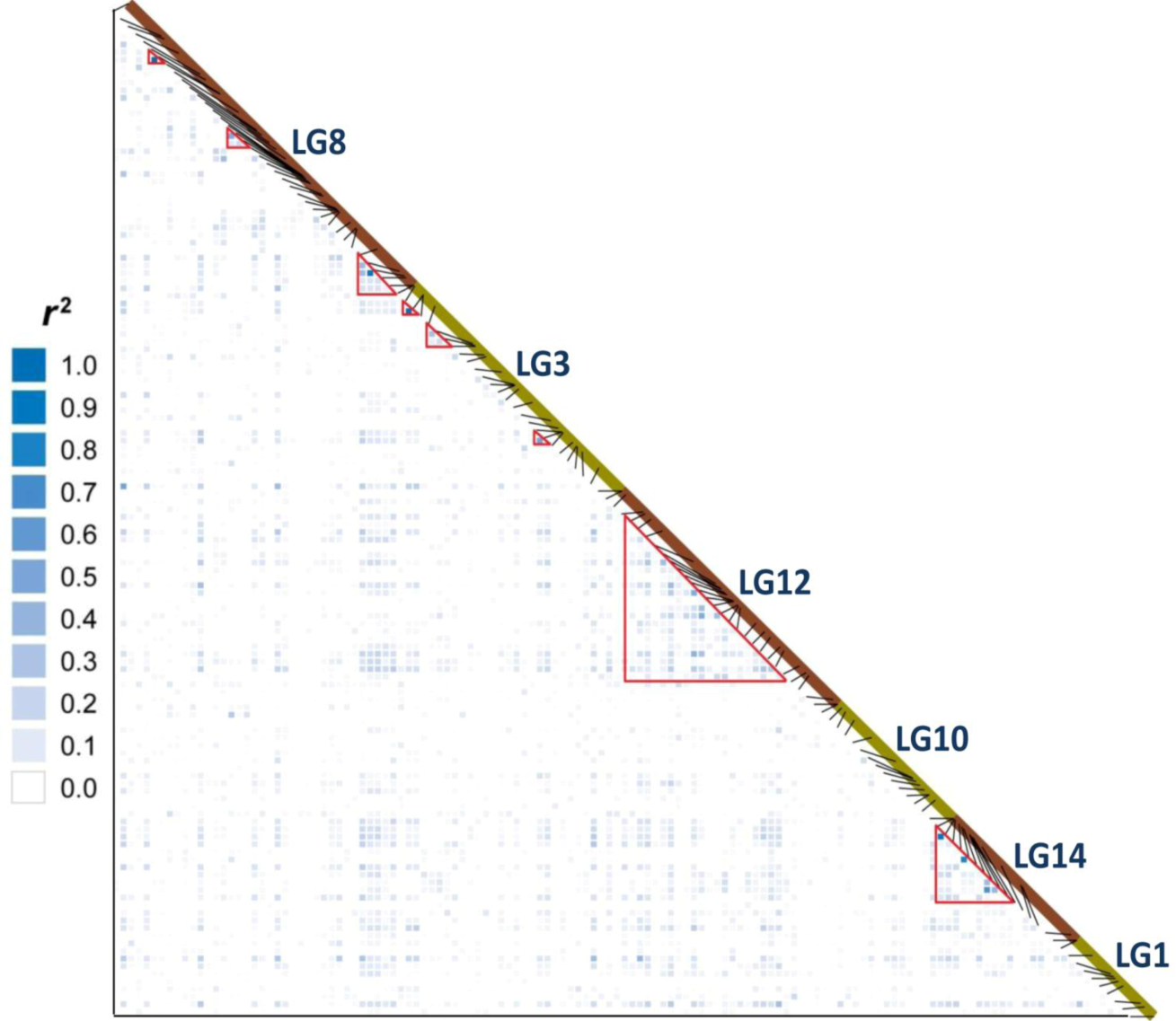


**Fig. S1** Linkage disequilibrium (LD) heat map of six linkage groups (LGs) in linseed. Red triangles highlight blocks of LD across LGs and the colored ruler indicates the strength of LD (*r*^2^)

**Online Resource 4**

**Fig. S2** Pairwise relative kinship estimates of the flax core collection based on 448 microsatellite markers

**Online Resource 5**

**
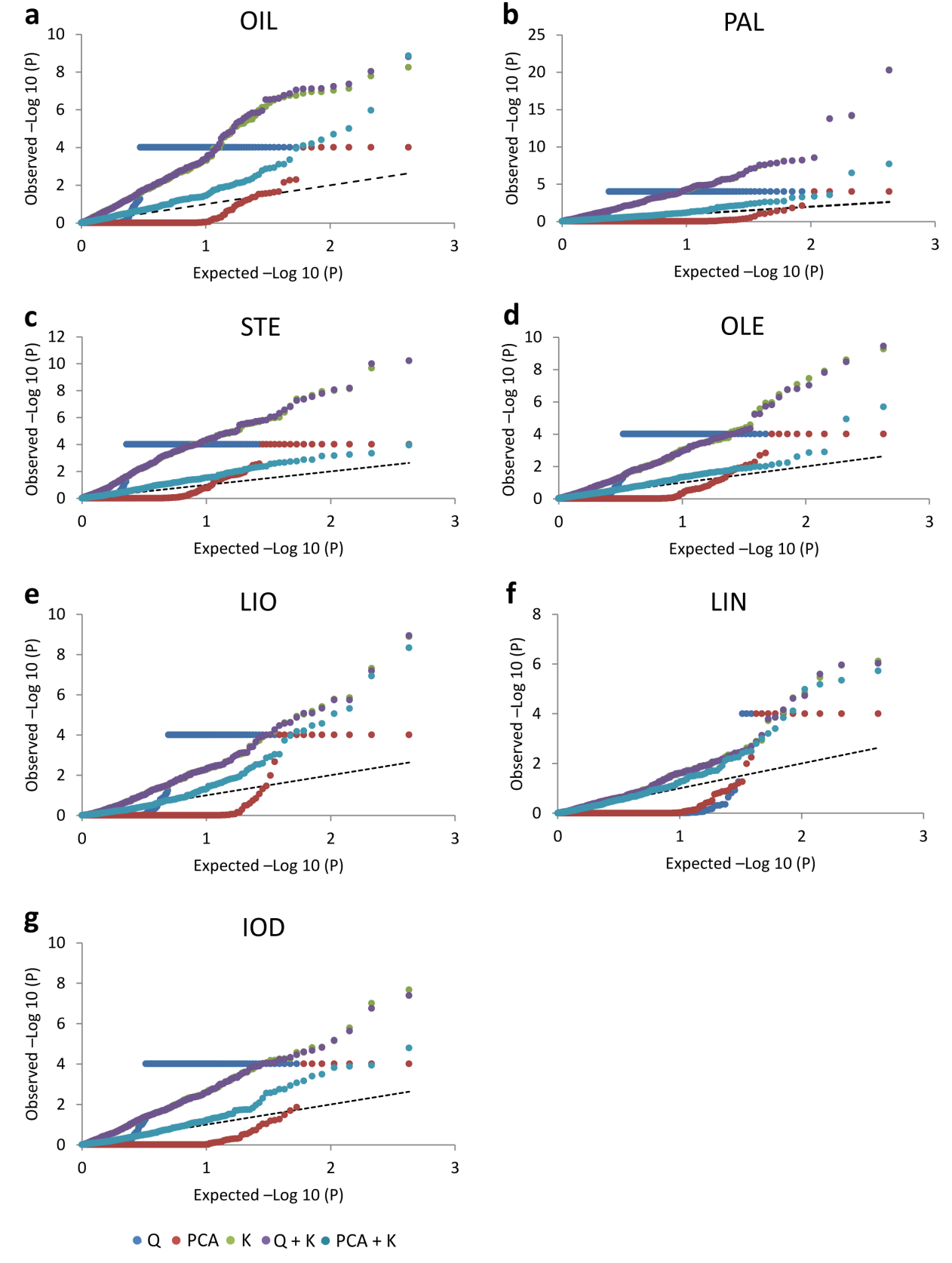
**

**Fig. S3** Cumulative probability-probability (P-P) plots of the observed -Log_10_ (P) values (y-axes) against the expected distribution (dotted diagonal line) of -Log_10_ (P) values (x-axes) for the general linear model (*Q*), the general linear model (PCA), the mixed linear model (*K*),the mixed linear model (*Q* + *K*) and the mixed linear model (PCA + *K*). **a** oil content (OIL) **b** palmitic acid content (PAL) **c** stearic acid content (STE) **d** oleic acid content (OLE) **e** linoleic acid content (LIO) **f** linolenic acid content (LIN) **g** iodine value (IOD)

**Online Resource 6**

**Table S3** Candidate QTL and associated markers with seven seed quality traits identified at either or both of the Manitoba (MB) and Saskatchewan (SK) locations

| Trait | Contig-Scaffold-Marker | LG | Position | LOC | -Log_10_ (P) | *R*^2^ (%) | Effect^a^ | Favorable allele (bp) |
| --- | --- | --- | --- | --- | --- | --- | --- | --- |
| OIL | c108-s305_Lu2649 | 8 | 18.92 | MB | 4.19 | 1.72 | 0.66* | 228 |
|  | **c31-s67_Lu181** | 9 | 31.34 | MB | 3.47 | 3.70 | 1.12* | 270 |
|  |  |  |  | SK | 3.65 | 8.31 | 1.40** | 270 |
|  | c31-s8_Lu2262 | 9 | 23.58 | SK | 3.50 | 1.77 | 1.69** | 186 |
|  | c77-s151_Lu519 | 9 | 32.54 | MB | 3.30 | 1.49 | 0.81* | 239 |
|  |  |  |  | SK | 3.32 | 4.61 | 0.96** | 239 |
|  | c0-s0_Lu926Bb | 12 | 32.87 | MB | 3.81 | 2.03 | 0.62* | 596 |
|  | Lu242 | - | - | MB | 3.30 | 2.83 | 1.53** | 324 |
|  | Lu401 | - | - | MB | 4.11 | 9.66 | 1.70** | 317 |
|  | Lu788 | - | - | MB | 3.41 | 1.07 | 0.92** | 280 |
| PAL | c7-s471_Lu2040 | 3 | 74.36 | MB | 6.83 | 1.41 | 0.30* | 147 |
|  |  |  |  | SK | 8.05 | 3.32 | 0.31* | 147 |
|  | c79-s540_Lu2534 | 7 | 5.79 | MB | 6.67 | 1.01 | 0.90** | 312 |
|  |  |  |  | SK | 6.60 | 2.09 | 0.71** | 312 |
|  | c38-s34_Lu2046 | 11 | 0.00 | MB | 3.10 | 8.44 | 1.01** | 152 |
|  | c214-s863_Lu2917a | 12 | 0.00 | MB | 3.29 | 4.24 | 0.35** | 215 |
|  | Lu681 | - | - | SK | 4.37 | 1.27 | 0.33* | 310 |
| STE | c222-s821_Lu943 | 1 | 149.99 | MB | 3.41 | 2.04 | 0.62** | 274 |
|  | c160-s260_Lu747b | 2 | 0.00 | SK | 3.40 | 1.03 | 0.96** | 261 |
|  | c400-s216_Lu3150 | 3 | 113.37 | MB | 3.62 | 8.34 | 1.17** | 366 |
|  | c118-s196_Lu558 | 3 | 122.39 | MB | 4.06 | 1.26 | 0.21* | 265 |
|  |  |  |  | SK | 3.37 | 5.91 | 0.24* | 265 |
|  | c171-s62_Lu1112 | 6 | 84.84 | MB | 3.40 | 9.18 | 0.39* | 270 |
|  | c79-s511_Lu2532 | 7 | 2.73 | MB | 3.64 | 4.92 | 1.64** | 270 |
|  |  |  |  | SK | 3.51 | 3.34 | 1.29** | 270 |
|  | **c175-s1216_Lu146** | 7 | 23.95 | MD | 3.32 | 13.2 | 1.01** | 354 |
|  |  | 7 | 23.95 | SK | 3.37 | 19.68 | 1.00** | 354 |
|  | c175-s1216_Lu151 | 7 | 23.99 | SK | 3.20 | 9.07 | 0.73** | 286 |
|  | c0-s635_Lu928 | 8 | 74.30 | SK | 3.49 | 1.51 | 1.01** | 243 |
|  | c32-s0_Lu2279 | 13 | 37.19 | MB | 3.51 | 3.63 | 0.79** | 210 |
|  | Lu316 | - | - | MB | 3.42 | 10.21 | 0.69** | 223 |
|  |  |  |  | SK | 3.40 | 3.79 | 0.49** | 223 |
|  | Lu319 | - | - | MB | 3.40 | 2.13 | 0.55** | 230 |
|  | Lu401 | - | - | MB | 3.63 | 1.04 | 0.51** | 317 |
| STE | Lu707 | - | - | MB | 3.54 | 9.26 | 0.92** | 538 |
| OLE | c82-s1491_Lu2564 | 6 | 64.09 | MB | 3.96 | 1.18 | 3.66** | 251 |
|  |  |  |  | SK | 5.41 | 1.94 | 2.72** | 251 |
|  | c82-s176_Lu2555 | 6 | 72.00 | MB | 4.24 | 3.97 | 2.45** | 217 |
|  |  |  |  | SK | 4.84 | 3.93 | 2.07** | 217 |
| LIO | **c729-s156_Lu3262** | 3 | 55.74 | MB | 5.99 | 8.34 | 0.93* | 195 |
|  |  |  |  | SK | 5.18 | 6.91 | 0.75* | 195 |
|  | **c0-s156_Lu64** | 3 | 60.88 | MB | 3.76 | 2.75 | 3.94** | 220 |
|  |  |  |  | SK | 4.12 | 2.88 | 3.62** | 220 |
|  | c16-s156_Lu373 | 3 | 64.44 | MB | 3.95 | 1.79 | 2.94** | 216 |
|  |  |  |  | SK | 3.80 | 1.02 | 2.73** | 216 |
|  | c202-s39_Lu41 | 5 | 57.36 | MB | 7.41 | 0.89 | 0.65* | 336 |
|  |  |  |  | SK | 6.41 | 0.82 | 0.62* | 336 |
|  | c30-s11_Lu164 | 5 | 57.89 | MB | 3.60 | 1.72 | 0.53* | 211 |
|  |  |  |  | SK | 3.50 | 1.93 | 0.54* | 211 |
|  | c436-s86_Lu296 | 7 | 40.77 | MB | 3.60 | 3.31 | 2.05** | 526 |
|  | c436-s86_Lu672 | 7 | 43.41 | MB | 3.82 | 2.72 | 2.10** | 197 |
|  | c108-s159_Lu585B | 7 | 53.67 | MB | 3.63 | 1.07 | 0.99* | 208 |
|  |  |  |  | SK | 3.50 | 1.11 | 0.93* | 208 |
|  | c281-s1851_Lu566 | 7 | 91.55 | MB | 5.11 | 1.08 | 1.19** | 214 |
|  |  |  |  | SK | 5.32 | 1.12 | 1.08** | 214 |
|  | c82-s617_Lu2561a | 8 | 28.95 | MB | 4.19 | 0.76 | 0.78** | 338 |
|  |  |  |  | SK | 3.67 | 0.89 | 0.70** | 338 |
|  | c46-s505_Lu2102 | 8 | 72.74 | MB | 7.73 | 2.96 | 0.77* | 241 |
|  |  |  |  | SK | 9.65 | 7.57 | 0.63* | 241 |
|  | c306-s98_Lu206b | 12 | 71.90 | MB | 4.39 | 1.07 | 0.56* | null |
|  |  |  |  | SK | 3.96 | 1.41 | 0.54* | null |
|  | c306-s98_Lu203b | 12 | 72.55 | MB | 4.03 | 0.93 | 0.51* | null |
|  |  |  |  | SK | 3.58 | 1.41 | 0.58* | null |
|  | **c306-s98_Lu765Bb** | 12 | 75.12 | MB | 5.05 | 4.76 | 0.97* | null |
|  |  |  |  | SK | 4.84 | 4.42 | 0.90* | null |
|  | Lu771 | - | - | MB | 3.60 | 2.99 | 1.17* | 230 |
|  |  |  |  | SK | 3.59 | 1.81 | 1.46** | 230 |
| LIN | **c729-s156_Lu3262** | 3 | 55.74 | MB | 4.17 | 3.68 | 1.17* | 195 |
|  |  |  |  | SK | 4.61 | 5.26 | 1.34* | 195 |
|  | c16-s156_Lu373 | 3 | 64.44 | MB | 3.50 | 1.80 | 2.10** | 216 |
| LIN |  |  |  | SK | 4.62 | 4.85 | 1.67** | 216 |
|  | c30-s11_Lu164 | 5 | 57.89 | MB | 3.63 | 2.65 | 1.81** | 211 |
|  |  |  |  | SK | 3.51 | 3.07 | 1.24** | 211 |
|  | **c202-s39_Lu41** | 5 | 57.36 | MB | 4.22 | 1.90 | 2.04** | 323 |
|  |  |  |  | SK | 4.81 | 3.45 | 1.54* | 323 |
|  | c108-s159_Lu585B | 7 | 53.67 | MB | 3.60 | 0.98 | 0.71* | 208 |
|  |  |  |  | SK | 3.51 | 1.32 | 0.79* | 208 |
|  | c281-s1851_Lu566 | 7 | 91.55 | MB | 3.60 | 0.92 | 0.64* | 214 |
|  |  |  |  | SK | 3.86 | 0.96 | 0.77* | 214 |
|  | c82-s617_Lu2561a | 8 | 28.95 | MB | 4.15 | 1.85 | 2.26** | 338 |
|  |  |  |  | SK | 4.83 | 1.78 | 2.04** | 338 |
|  | c46-s505_Lu2102 | 8 | 72.74 | MB | 5.32 | 3.63 | 0.74* | 241 |
|  |  |  |  | SK | 6.85 | 5.07 | 0.78* | 241 |
|  | c141-s641_Lu2746 | 10 | 87.69 | MB | 3.60 | 4.31 | 2.40** | 406 |
|  |  |  |  | SK | 3.50 | 2.55 | 1.73** | 406 |
|  | c306-s98_Lu206b | 12 | 71.90 | MB | 3.84 | 0.93 | 0.76* | 214 |
|  |  |  |  | SK | 4.20 | 0.92 | 0.79* | 214 |
|  | c306-s98_Lu203b | 12 | 72.55 | MB | 3.60 | 1.20 | 0.79* | 214 |
|  |  |  |  | SK | 3.69 | 1.31 | 0.72* | 214 |
|  | **c306-s98_Lu765Bb** | 12 | 75.12 | MB | 3.60 | 1.18 | 0.92* | null |
|  |  |  |  | SK | 4.40 | 2.33 | 0.87* | null |
| IOD | c28-s475_Lu2247 | 2 | 50.86 | MB | 3.96 | 2.21 | 9.8** | 285 |
|  | c729-s156_Lu3262 | 3 | 55.74 | SK | 4.43 | 1.43 | 4.99** | 195 |
|  | c82-s176_Lu2555 | 6 | 72.00 | MB | 3.84 | 4.83 | 8.58** | 214 |
|  |  |  |  | SK | 8.85 | 5.13 | 6.78** | 214 |
|  | c82-s617_Lu2561a | 8 | 28.95 | MB | 3.61 | 2.12 | 5.90** | 338 |
|  | c46-s505_Lu2105 | 8 | 72.13 | MB | 3.60 | 2.28 | 13.60** | 226 |
|  | c46-s505_Lu2102 | 8 | 72.74 | SK | 4.23 | 9.35 | 9.31** | 241 |
|  | **c0-s635_Lu928** | 8 | 74.30 | MB | 3.89 | 3.03 | 9.66** | 247 |
|  |  |  |  | SK | 3.49 | 3.53 | 9.30** | 247 |
|  | c141-s641_Lu2746 | 10 | 87.69 | MB | 3.60 | 1.42 | 5.34** | 406 |
|  | Lu720 | - | - | SK | 3.41 | 0.89 | 7.76** | 321 |

^a^Effect of favorable alleles represents the increment in percentage of FAs. For IOD the effect represent iodine value in units

Significance of the allelic effects tested by Kruskal-Wallis non-parametric test **P* < 0.01; ***P* < 0.001

Markers in bold script represent the candidate QTL

**Online Resource 7**

**
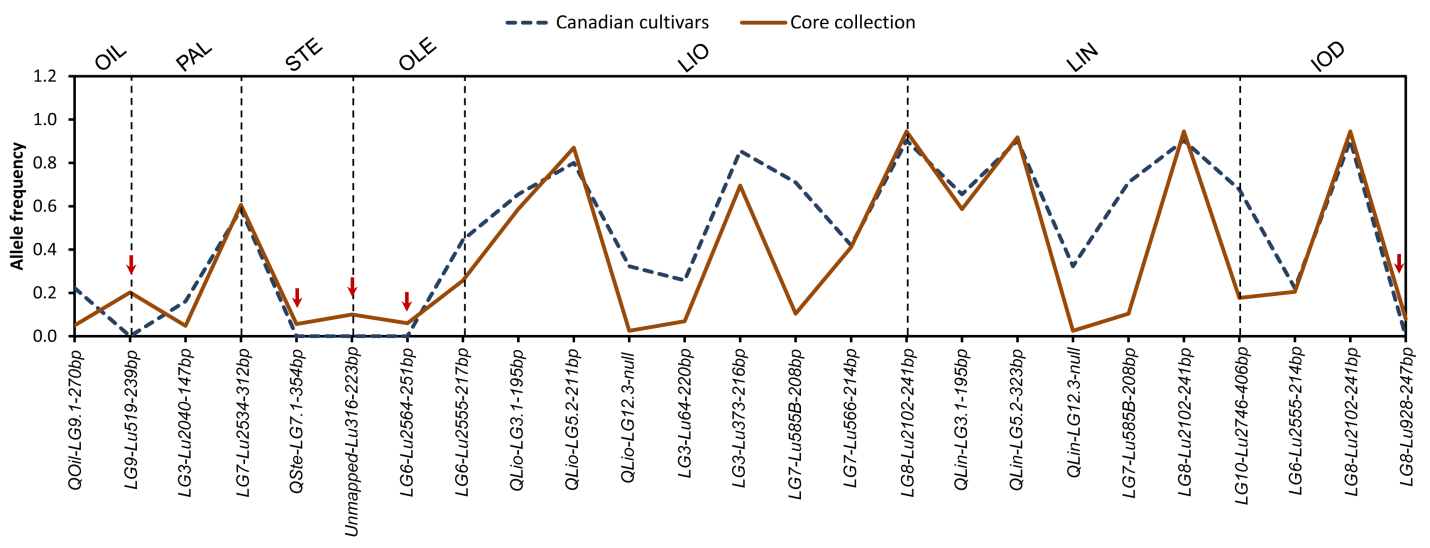
**

**Fig. S4** Comparison of the frequency of favourable QTL/marker alleles across seven quality traits in 30 linseed Canadian cultivars and the remaining 377 accessions of the flax core collection. Arrows indicate absent alleles in Canadian germplasm
